# Pollen diet diversity across bee lineages varies with lifestyle rather than colony size

**DOI:** 10.1093/jisesa/ieae023

**Published:** 2024-03-05

**Authors:** Kedar Devkota, Charles F dos Santos, Patrick D Souza-Santos, Jenifer D Ramos, Alex Otesbelgue, Binayak Prakash Mishra, Eduardo A B Almeida, Betina Blochtein

**Affiliations:** Faculty of Agriculture, Agriculture and Forestry University, Rampur, Chitwan, Nepal; Departamento de Fitossanidade, Faculdade de Agronomia, Universidade Federal do Rio Grande do Sul, Av. Bento Gonçalves, 7712, Porto Alegre, Rio Grande do Sul, 91540-000, Brazil; Departamento de Biologia, Laboratório de Biologia do Desenvolvimento de Abelhas, Faculdade de Filosofia, Ciências e Letras de Ribeirão Preto, Universidade de São Paulo, Av. Bandeirantes, 3.900, Ribeirão Preto, São Paulo 14040-901, Brazil; Embrapa Meio Ambiente, Rodovia SP-340, Km 127.5, Jaguariúna, São Paulo 13918-110, Brazil; Programa de Pós-graduação em Ecologia, Department of Zoology, Bird and Mammal Evolution, Systematics and Ecology Lab, Instituto de Biociências, Universidade Federal do Rio Grande do Sul, Av. Bento Gonçalves, 43435, Porto Alegre, Rio Grande do Sul 90650-001, Brazil; Faculty of Agriculture, Agriculture and Forestry University, Rampur, Chitwan, Nepal; Laboratório de Biologia Comparada e Abelhas, Departamento de Biologia, Faculdade de Filosofia, Ciências e Letras de Ribeirão Preto, Universidade de São Paulo, Ribeirão Preto, São Paulo 14040-901, Brazil; Mais Abelhas Consultoria Ambiental Co., Avenida Ipiranga, 6681, Partenon, Porto Alegre, Rio Grande do Sul, CEP 90619-900, Brazil

**Keywords:** cooperative behavior, diet breadth, eusociality, phylogeny, pollen

## Abstract

The shift to a pollen diet and the evolution of more highly organized societies, i.e., eusocial, were key milestones in bee diversification over their evolutionary history, culminating in a high dependence on feeding broods with a large variety of floral resources. Here, we hypothesized that obligatory eusocial bees have a wider diet diversity than their relatives with solitary lifestyles, and this would be related to colony size. To test both hypotheses, we surveyed diet breadth data (palynological analysis) based on the Shannon–Wiener index (Hʹ) for 85 bee taxa. We also obtained colony size for 47 eusocial bee species. These data were examined using phylogenetic comparative methods. The results support the generalist strategy as a derived trait for the bee taxa evaluated here. The dietary diversity of eusocial bees (Hʹ: 2.1, on average) was 67.5% higher than that of noneusocial bees (Hʹ: 1.21, on average). There was, however, no relationship between diet breadth and colony size, indicating that smaller colonies can harvest a pollen variety as diverse as larger colonies. Taken together, these results provide new insights into the impact of lifestyle on the diversity of collected pollen. Furthermore, this work sheds light on an advantage of living in more highly structured societies irrespective of the size of the colony.

## Graphical Abstract

**Graphical Abstract:**
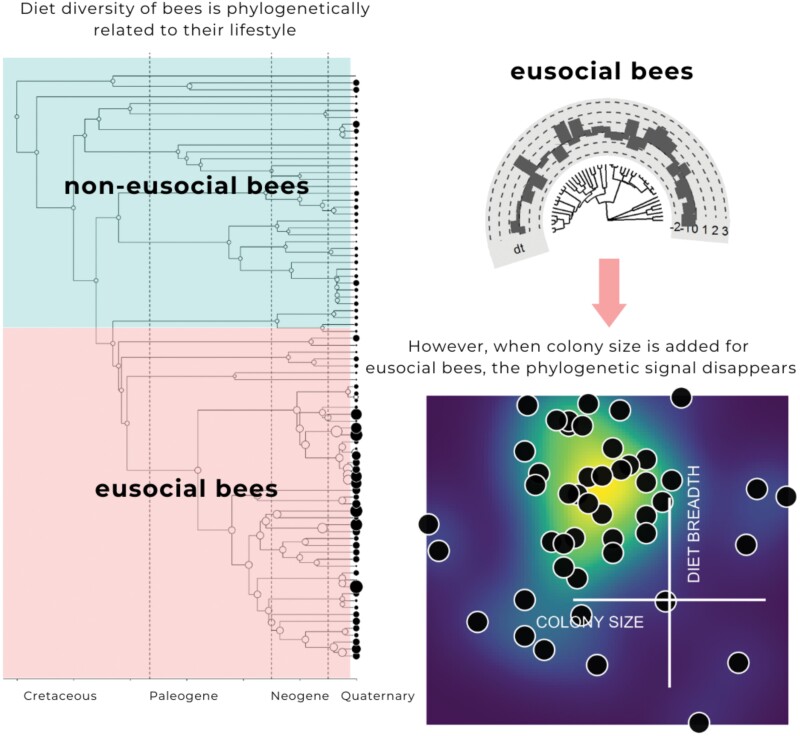

## Introduction

Bees (Hymenoptera: Anthophila) abandoned the predatory lifestyle of their close relatives, such as the sand wasps (Crabronidae) or digger wasps (Sphecidae) (Branstetter et al. 2017, Peters et al. 2017). The transition from being carnivorous to relying on pollen as a protein source occurred during the Cretaceous period, approximately 124–111 million years ago (Branstetter et al. 2017, Peters et al. 2017). This period also witnessed a remarkable diversification of bees and vascular plants, particularly the eudicot angiosperms (Cardinal and Danforth 2013, Peters et al. 2017). Subsequently, strict eusociality, characterized by a complex social organization, emerged in the corbiculate clade (bumblebees, honeybees, and stingless bees) around 65–87 million years ago (Cardinal and Danforth 2011). Thus, the shift to a pollen-based diet and the development of sophisticated social structures were crucial milestones in the evolutionary history of bees, contributing to their diversification over time.

Bees can exhibit a range of hierarchical lifestyles, including solitary, subsocial, communal, semi-social, quasi-social, parasocial, and eusocial arrangements (Wilson 1971, Crespi and Yanega 1995, Costa and Fitzgerald 2005). These terms describe how parents, usually reproductive females, engage in care, communication, task-sharing with their offspring, and how individuals within the same species display varying degrees of nest fidelity and aggregative tendencies (Wilson 1971, Wcislo and Cane 1996, Costa and Fitzgerald 2005).

The eusocial lifestyle offers a suite of interconnected characteristics, including (i) overlapping generations, (ii) cooperative brood care, (iii) division of labor through castes such as queens and workers, and occasionally (iv) the presence of soldier castes or defender morphs (Michener 1969, Wilson 1971, Crespi and Yanega 1995, Costa and Fitzgerald 2005, Wilson and Holldobler 2005). Additional life-history traits of social organization have also been more recently proposed (Cardinal and Danforth 2011). In these eusocial societies, the reproductive female, known as the queen, is able to dedicate all her energy to egg-laying, while her daughters undertake specialized roles such as defense and foraging (Michener 1969, 2007). The exception would be of bumble bees in temperate regions where colonies are annual and hibernated queens emerge in the spring, find a location for their nest, and forage until the first set of workers emerge at which point the queen can concentrate on egg-laying inside the nest.

Bees that exhibit less cooperative behaviors, such as solitary, communal, sub-, and semi-social arrangements, find a nest independently, and forage and store provisions for their offspring without the assistance of others (Wcislo and Cane 1996, Michener 2007, Shell and Rehan 2018). Once these bees supply the nest cavities with their broods (offspring), they may fill out more than one cavity throughout their lifespan or then either abandon their brood or perish, resulting in minimal or no contact with their offspring or conspecifics (Wcislo and Cane 1996, Michener 2007, Shell and Rehan 2018). Furthermore, their nests are rarely defended against predators or parasites (Wcislo and Cane 1996, Michener 2007, Shell and Rehan 2018).

Given the substantial differences between the lifestyles of eusocial bee taxa and noneusocial lineages, it is reasonable to speculate that the dietary breadth of eusocial bees would reflect their highly intricate societies, characterized by elaborate communication systems and a large number of individuals. In this regard, a broader diet breadth might be associated with the necessity to forage across a diverse range of plant taxa to sustain the nutritional needs of the numerous individuals continuously emerging within these populous societies. Moreover, eusocial bees rely on a consistent food supply to support their perennial colonies, typically consisting of a single queen and hundreds or even thousands of workers. Consequently, it is plausible to suspect that a larger foraging workforce would be inclined to exploit a wider variety of plant taxa, benefiting from the presence of multiple bees specialized in this task. Ultimately, the cooperation and interactions among individuals within a eusocial colony may facilitate enhanced access to food resources.

Hence, if we assume that eusocial bees possess a greater dietary diversity compared to less complex and structured lineages, it follows that clades with more populous colonies would exhibit higher levels of food diversification, owing to the increased availability of foragers. The scale of population variation among colonies of eusocial bee species can vary significantly, potentially reaching an order of magnitude of 1 × 10^5^ in terms of the observed number of bee workers (see Supplementary Material S1). Therefore, we hypothesize that a correlation exists between diet breadth and colony size in bees.

In the present study, our hypothesis posits that obligatory eusocial lineages of bees exhibit a broader diet breadth compared to their noneusocial relatives. This is attributed to the presence of multiple bees engaged in cooperative foraging in eusocial conditions, while in noneusocial situations, reproductive females experience limited or no cooperation. Additionally, we anticipate that colonies of eusocial bees with higher population sizes will demonstrate greater dietary diversity. To achieve our primary objectives, we employed phylogenetic comparative methods, aiming to (i) estimate the historical diversification of diet breadth among the sampled bees, (ii) compare the dietary diversification between noneusocial and eusocial lineages, and (iii) investigate the relationship between diet breadth and colony size.

## Methods

### Noneusocial vs. Eusocial Bees

In this study, we have collectively classified noneusocial bee taxa as those in which reproductive females independently construct nests, forage, and store food for their offspring, with limited or no cooperation among individuals residing in the same nests. Conversely, eusocial bees have been categorized as lineages that irreversibly exhibit the combined attributes of eusociality previously described. Therefore, bee taxa that can exhibit facultative eusociality, such as certain sweat and orchid bees, have been classified as noneusocial. This classification is based on the fact that eusociality is not obligatory in these taxa and is primarily associated with specific ecological traits, such as the number and temporal sequence of individuals emerging within nests and availability of nesting space (Soro et al. 2010, Andrade-Silva and Nascimento 2012, Boff et al. 2015, Davison and Field 2018, Shell and Rehan 2018).

### Pollen Diversity as a Proxy of Diet Breadth

The analysis of food diversity in bees has been a subject of frequent investigation through palynology, which involves the examination of pollen samples found in pollen loads of foragers, pollen pots, brood cells provisioned by reproductive females, and even fecal pellets (Vit et al. 2018). As a whole, when examining eusocial bees, researchers commonly gather samples from approximately 5–10 returning pollen foragers, although this information may not always be provided in the original articles. The collection process involves the use of entomological nets positioned in front of nest entrances to capture and analyze the loads carried by the bees. In the case of noneusocial bees, most studies typically involve the collection of pollen grains found in individual brood cells, which may contain either food or feces. Furthermore, the predominant method for quantifying plant species/types involves researchers randomly counting between 200 and 400 pollen grains on 3–5 slides per sampling (see original articles for details in Supplementary Material S1).

To assess the range of diets in bees, we conducted a comprehensive review of published literature utilizing palynological data as a proxy for dietary diversity. Our search encompassed the terms “bees AND palynology,” “bees AND pollen,” and “bees AND Shannon index” in databases such as ISI Web of Science (https://www.webofknowledge.com), Google Scholar (https://scholar.google.com/), and Scielo (https://scielo.org). In cases where the original articles did not provide the Shannon–Wiener diversity index (*H*ʹ), we utilized the information presented in tables by the authors to calculate it. The references utilized in this study are provided in Supplementary Material S1.

### Shannon–Wiener Diversity Index as a Metric for Estimating Diet Breadth

The Shannon–Wiener index, initially developed as an information theory concept (Shannon and Weaver 1949), serves to measure the level of entropy or uncertainty in strings of text within a message that can be encoded, compressed, and subsequently recovered with minimal chances of error. The fundamental idea behind the Shannon–Wiener index is that the greater the diversity and relative abundance of letters within the information content, the more challenging it becomes to predict the next letter in the sequence accurately.

In the field of ecology, the Shannon–Wiener index is employed to quantify the uncertainty associated with predicting randomly occurring taxa within a dataset. As such, the Shannon–Wiener index, often employed in ecological studies, serves as a robust metric for assessing the biodiversity of a given ecosystem. This index takes into consideration not only the sheer number of species within the ecosystem but also factors in their relative abundances, providing a comprehensive measure that reflects both species richness and evenness in the ecological community. High diversity, as indicated by a higher Shannon–Wiener index, suggests a greater amount of information or a larger number of potential scenarios. The Shannon–Wiener index can be calculated using the following formula:


$$\begin{array}{l} H\overset{´}{\;}\;=\;\text{-}\sum_{i=1}^{s}p_{i}*ln\;(p_{i}) \end{array}$$

where *p*_*i*_ is the proportion of individuals found in the _*i*_th species (_*i*_)—relative abundance (*n*_*i*_/*N*; *n*_*i*_ is the number of individuals in species [*i*], and *N* is the total number of individuals over all species), ln is the natural logarithm (*e* = 2.711828), *Σ* is the sum of the calculations, and *s* is the number of species observed. Since the Shannon–Wiener diversity index is sensitive to rarer species, it is often used in conservation projects (Magurran 1988, Spellerberg and Fedor 2003). If there was more than one value for that index, then we extracted the average value for a particular bee species. It is noteworthy to mention that in the studies surveyed here, the collection of pollen grains for subsequent diversity analysis was carried out in various locations (biomes, countries) and at different time intervals, ranging from a few days to weeks and sometimes spanning different seasons. The diverse range of data obtained poses a bottleneck in our analysis. Nevertheless, encountering such variability is a common challenge when employing phylogenetic comparative methods. Finally, differences in sampling methods among bee groups may impact the *H*ʹ. This index is sensitive to changes in species abundance, and discrepancies in sampling efforts may introduce biases or lead to underestimation or overestimation of diversity.

### Colony Size

Following the completion of our literature search on pollen diversity, we specifically focused on eusocial bee species for which data on diet breadth were available. We then proceeded to investigate the corresponding colony sizes for these species. In cases where multiple values for the number of bees within nests were reported for a particular bee species, we extracted the average colony size value. To gather information on colony size, we conducted searches using three relevant terms: ‘colony size’, ‘number of bees’, and ‘bee population’. The same online databases described above were utilized for this purpose. The references used in this study are provided in Supplementary Material S1.

### Diet Breadth Based on Phylogeny, Sociality, and Colony Size

To date, only a limited number of palynological analyses have assessed the diet breadth of bee species with diverse lifestyles, with a majority of studies focusing on Apidae. Therefore, to ensure robustness in our analyses, we established 3 criteria for including species in our study: (i) availability of diet breadth data, (ii) reported lifestyle information, and (iii) colony size information if the species was eusocial. Consequently, we compiled a reference list comprising data from 85 bee species (38 noneusocial and 47 eusocial) based on their diet breadth (*H*ʹ). The same procedure was employed to gather information on diet breadth and colony size for eusocial taxa.

For the comparative analysis of these 85 species, we utilized a phylogenetic framework based on the findings of Bossert et al. (2019) and divergence time estimates by Cardinal and Danforth (2013), supplemented by estimates from Meliponini (Rasmussen and Cameron 2010), Bombini (Hines 2008), Centridini (Martins and Melo 2016), and Euglossini (Ramírez et al. 2010). Most branch lengths were estimated proportionally to time, except for the relationships among Emphorini, *Tetrapedia*, *Frieseomelitta*, and *Scaptotrigona* species, as well as some lineages of *Melipona*. In these cases, closely related species were clustered within their respective clades, and a divergence time close to zero was assigned, resulting in soft polytomies that reflected our uncertainty about their specific relationships (Garland and Díaz-Uriarte 1999). Subsequently, the complete 85-species chronogram was pruned to include only the 47 terminal species recognized as obligate eusocial (see Supplementary Material S1).

The estimation of ancestral dietary diversity was conducted by mapping *H*ʹ values onto different nodes of the bee phylogeny, using extant species as a basis. This reconstruction was performed using the anc.ML function in the R package “phytools” (Revell, 2012), employing maximum likelihood estimation under the Brownian model with 99,999 simulations. To assess the similarity of closely related bee species in terms of *H*ʹ values, indicative of shared evolutionary history, we calculated the phylogenetic signal using Pagel’s λ with the phylosig function in the R package “phytools” (Revell 2012). A value of λ equal to 1 indicates that changes in traits can be explained solely by the phylogeny, following a pure Brownian motion model of evolution (strong phylogenetic signal) (Kamilar and Cooper 2013). Conversely, a value of λ equal to 0 suggests that trait evolution has occurred independently of the phylogeny. Additionally, λ can exceed 1, indicating a higher rate of trait evolution at the root compared to the tips of the phylogeny (Kamilar and Cooper 2013).

To address the issue of statistical nonindependence among species, we employed phylogenetic comparative methods (Felsenstein 1985, Grafen 1989) in our statistical analysis. These methods allow for robust statistical inference by accounting for shared evolutionary history, enabling us to explore the origins and maintenance of trait differences across the phylogeny of organisms (Felsenstein 1985, Grafen 1989, Harvey and Pagel 1991, Pagel 1994). By applying phylogenetic comparative methods, we can investigate how biological and ecological traits are associated with patterns and processes of trait evolution (Felsenstein 1985, Grafen 1989, Harvey and Pagel 1991, Pagel 1994). Ancestral state reconstruction, a key component of these methods, allows us to estimate the phenotypic traits at ancestral nodes in the phylogenetic tree (Omland 1999, Revell 2014). This enables us to map the diversification of traits throughout evolutionary history and infer their most likely origins based on the trait values observed in extant descendants (living taxa) (Omland 1999, Revell 2014). As we shall see, certain species of stingless bees significantly influenced the average *H*ʹ, indicating considerable variation within each category. To assess further into the dietary diversity among eusocial bees (stingless bees, bumblebees, and honeybees), we employed the coefficient of variation (CV), a key metric offering a standardized, percentage-based measure of relative variability in datasets.

To account for the nonindependence of closely related species and investigate the relationship between *H*ʹ and colony size, we utilized phylogenetic generalized least squares (PGLS) models (Grafen 1989). First, we applied a PGLS model to compare the *H*ʹ (dependent variable) between noneusocial and eusocial bee groups (predictor variables). Considering the discrete biological traits of bee groups, we incorporated the within-group correlation structure using the lambda parameter (λ). The PGLS model was fitted using the gls function in the R package “nlme” (Pinheiro et al. 2020). Similarly, we employed a second PGLS model to examine the relationship between diet breadth and colony size. However, since colony size and diet breadth in eusocial bees span a wide range of values, both continuous traits were log-transformed to facilitate meaningful comparisons. In addition, we performed phylogenetically independent contrasts using the function pic from the ape package (Paradis and Schliep 2019) to specifically extract the correlation between diet diversity and colony size for eusocial bees. All statistical analyses were conducted using the R programming language (Ihaka and Gentleman 1996, R Core Team 2021).

## Results

**Fig. 1.**
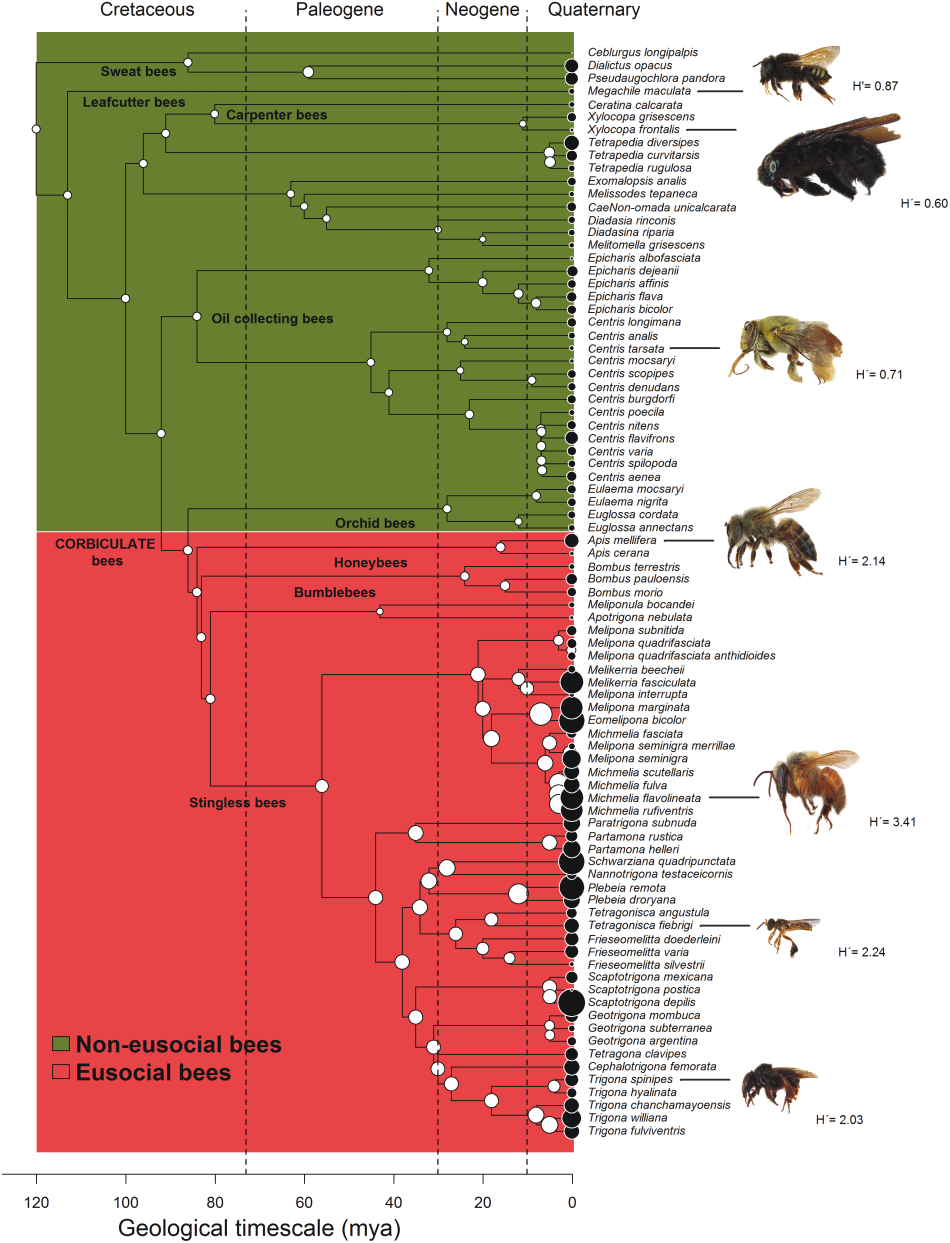
Reconstruction of diet diversity throughout bee phylogeny (85 species) from the ancestral state to the contemporary era. This phylogenetic reconstruction is based on previously published phylogenetic hypotheses (for details, refer to the Materials and methods section), showing the evolutionary history of diet width as measured by the Shannon–Wiener index, *H*ʹ (palynological analysis) across bee phylogeny. The white points over each node indicate the maximum likelihood of ancestral state reconstruction under a Brownian evolution model inferred after 99,999 simulations (estimated *H*ʹ), while black points over the tip labels show the current *H*ʹ. The pictures of bees illustrating phylogeny here were selected since they were available and, therefore, drawn from specimens from the scientific collection (Entomology Lab) of the Science and Technology Museum of Pontifical Catholic University, Rio Grande do Sul, Brazil. Shadowed rectangles denote those noneusocial clades vs. (obligatory) eusocial clades. The diet breadth (*H*ʹ) besides bee’s images was highlighted only for illustration. Original data were retrieved from the literature shown in Supplementary Information S1.

The common ancestor of exhibited a relatively low dietary diversity (*σ*² = 0.040, *H*ʹ ancestor = 1.20), and the analysis revealed a significant, albeit moderate, phylogenetic signal (Pagel’s λ = 0.28, LR = 13.4, *P* < 0.001) in relation to diet breadth. The diet breadth of noneusocial bees (average *H*ʹ noneusocial bees = 1.21) has remained relatively unchanged compared to the ancestral diet breadth (*H*ʹ ancestor = 1.20). In contrast, eusocial bees exhibited a notable increase in diet breadth (average *H*ʹ eusocial bees = 2.01), representing a 67.5% expansion from the ancestral level (*H*ʹ ancestor = 1.20). However, the higher mean for eusocial bees is mostly driven by some species of stingless bees (Fig. 1).

**Fig. 2.**
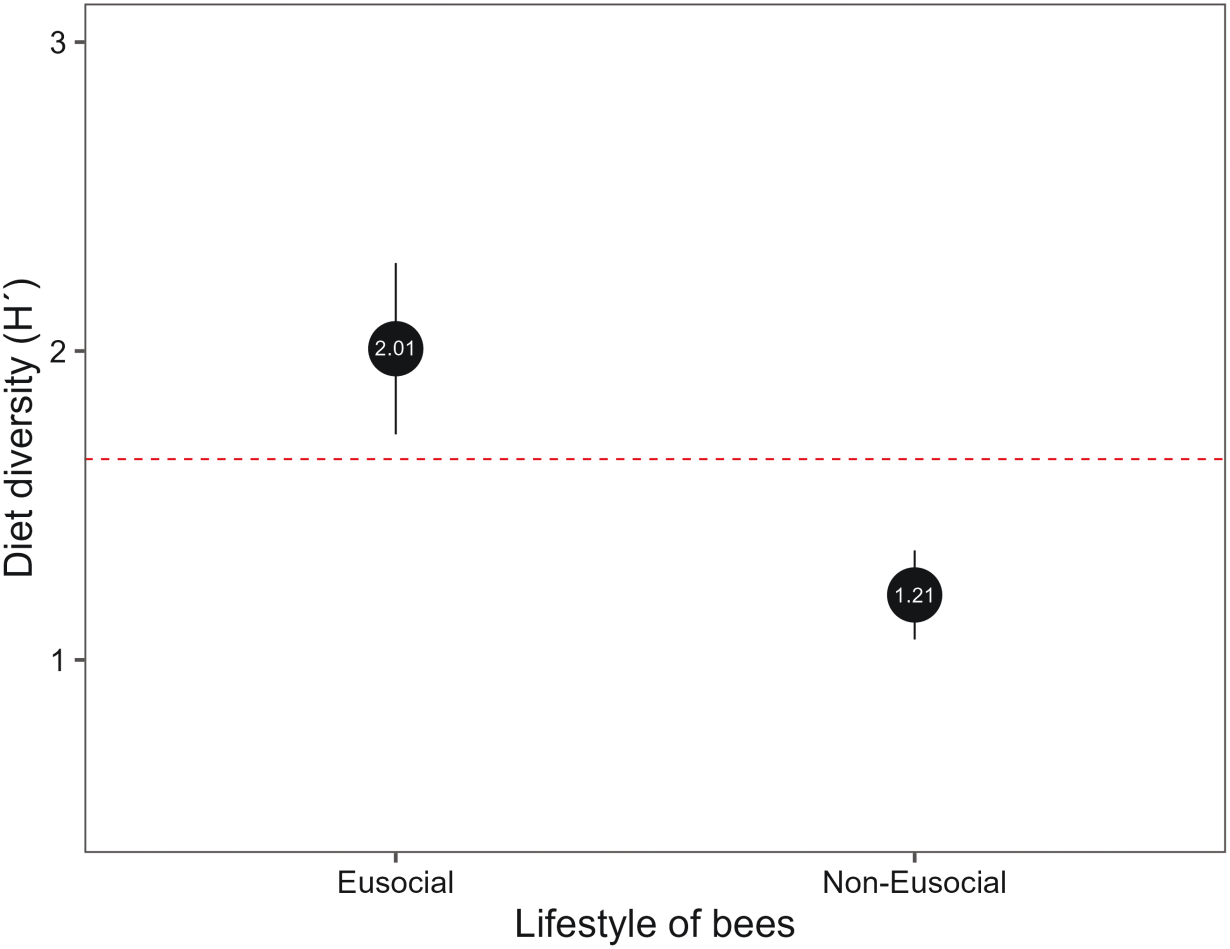
Comparison of diet width (Shannon–Wiener index, *H*ʹ) between noneusocial and eusocial bees after PGLS. Black points indicate the average value of *H*ʹ; vertical black lines show the confidence intervals (95%); the horizontal dashed line exhibits the overall mean (*H*ʹ = 1.69) calculated from all bee taxa combined.

The phylogenetic relationship among bee lineages contributes to the observed variations in diet breadth (PGLS, *F*_(1,83)_ = 11.2, *P* = 0.001, Fig. 2), suggesting that shared evolutionary history plays a role in shaping dietary differences among bee species. However, our dataset is notably enriched with stingless bee species, which may introduce biases to the results. Therefore, with the availability of more data on nonstingless bee species, further analysis could be undertaken to assess whether additional information may substantiate our findings. Our findings on the CV indicate that honeybees demonstrated the highest variability (75.5%), while stingless bees exhibited a moderate level of variability (45.6%), and bumblebees displayed a comparatively lower variability (31.7%). These results imply potential challenges in accurately predicting the dietary diversity of these bee groups.

**Fig. 3.**
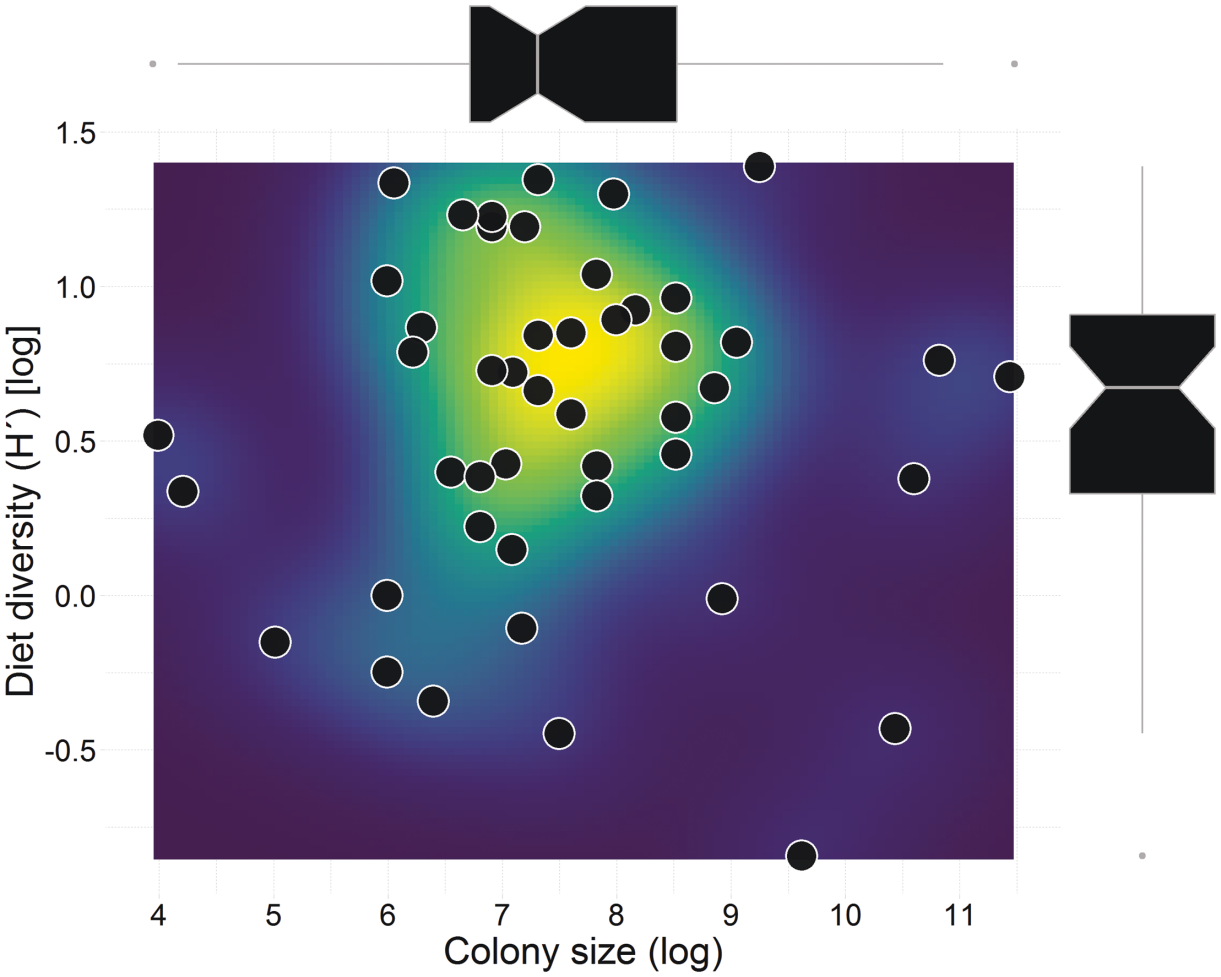
PGLS, dietary diversity vs. colony size: Relationship between diet breadth (Shannon–Wiener index, *H*ʹ) and colony size of eusocial bees. The points on the graph represent the observed values, which have been log-transformed using the natural logarithm. Box plot: the box = first and third quartiles, whiskers = the min and max range of variation, median (white line) = second quartile. Notched box plot: the box = first and third quartiles, whiskers = the min and max range of variation, median (white line) = second quartile. The notches extend 1.58 × IQR/sqrt(*n*). IQR refers to the interquartile range or distance between the first and third quartiles. Hence, the notches display the approximate confidence interval (95%) for comparing medians. The raster colors depict levels of data aggregation, from low count data to high count data.

Interestingly, contrary to our initial prediction, we did not find evidence to support that eusocial bee species residing in larger colonies possess a wider diet compared to those in smaller colonies (PGLS, *F*_(1,44)_ = 0.01, *P* = 0.88, Fig. 3). There was no correlation between diet breath and colony size (*t* = -0.50, df = 44, *P* = 0.61, *r* = −0.07). This unexpected result suggests that factors other than colony size may influence the dietary diversity of eusocial bees.

## Discussion

This study provides valuable insights into the evolution of dietary diversity in bees, particularly in relation to their pollen-feeding breadth, which serves as a crucial protein source for these insects, while providing other nutrients. The findings support the notion that the generalist strategy observed in the surveyed bee species, where they consume a wide range of pollen types, is a trait that evolved from a more specialized ancestral diet. This observation is consistent with previous studies (Danforth et al. 2013) and indicates that the transition toward a generalist feeding strategy occurred approximately 100 million years ago during the early Cretaceous period. Overall, our findings highlight the impact of phylogenetic relatedness on dietary diversity among bee lineages and shed light on the contrasting patterns observed between eusocial and noneusocial bees.

The ancestors of the bee species included in this study exhibited a dietary breadth similar to that of current noneusocial bees. This coincided with a period when bees had recently shifted from relying on animal protein sources to pollen as their primary source of nutrition (Branstetter et al. 2017, Peters et al. 2017). Importantly, this transition was accompanied by the diversification of both bees and angiosperms, particularly the eudicot angiosperms (Cardinal and Danforth 2013, Peters et al. 2017).

Our analysis also revealed a distinctive pattern where extant noneusocial bee taxa exhibited only slight modifications in their diet breadth, whereas eusocial bees displayed a notable expansion in their dietary preferences. The presence of a moderate phylogenetic signal suggests that phylogenetic relationships among the 85 bee species evaluated in our study have influenced the observed variation in pollen diet diversity. While other environmental and evolutionary factors may also contribute to the maintenance and diversification of this trait, the close relatedness of these bee species appears to be a relevant variable explaining their dietary breadth.

The evolution of eusociality, characterized by a complex social organization with a single reproductive female and several workers that transition into foragers, arose within the corbiculate clade encompassing bumblebees, honeybees, and stingless bees, around 65−87 million years ago (Cardinal and Danforth 2011). This social lifestyle likely facilitated or even enforced dietary diversification in eusocial bees compared to their noneusocial counterparts. The efficient communication system of eusocial bees enables the recruitment of multiple nestmates to exploit a wide range of available pollen resources in their environment (Nieh 2004, Dornhaus and Chittka 2005, Dornhaus et al. 2006). Additionally, morphological adaptations have played a role in expanding the food variety of eusocial bees. One notable adaptation is the transition from hairy hind legs to corbiculae, which are glabrous and concave structures on the hind tibiae that allow for efficient transport of pollen mixed with floral oil (Martins et al. 2014). This innovation in the Mid- to Late Cretaceous period, approximately 100–66 million years ago (Cardinal and Danforth 2013, Martins et al. 2014), coincided with the dominance of angiosperms (flowering plants that heavily rely on bees for pollination) in terms of species diversity (Cardinal and Danforth 2013).

Therefore, these highlights shed light on the factors contributing to the dietary diversification observed in eusocial bees. The evolution of their social structure, communication abilities, and specialized morphological features has likely played a significant role in facilitating their access to a broader range of food resources. This adaptive radiation of eusocial bees coincided with the rise of angiosperms, highlighting the mutualistic relationship between bees and flowering plants.

Regarding dietary diversity, although stingless bees displayed a moderate level of variability (45.6%) compared to honeybees (75.5%) and bumblebees (31.7%) within eusocial bees, they constituted almost half (47% of 85) of all taxa surveyed in our study. Consequently, stingless bees appear to significantly contribute to elevating the overall dietary diversity of eusocial bees. The assessed stingless bees exhibit diverse ecological attributes, with some nesting underground (e.g., Schwarziana and Geotrigona), others inside tree hollows (e.g., Frieseomelitta, Tetragonisca, Melipona), and others found in arboreal termite nests (e.g., *Partamona rustica*), or even nesting quite high above the ground in tree tops. While acknowledging the possibility of an overestimation of dietary variety due to these stingless bees, we propose that future analyses explore the congruence or divergence in the ecology of different stingless bee species as more data becomes available in the coming years.

Our analysis uncovered an intriguing finding: the foraging strategy of eusocial bees does not necessarily correlate with the size of their colonies. Surprisingly, we observed that small- to moderate-sized colonies can harvest a diverse array of resources comparable to their larger counterparts. This implies that even colonies with only a few hundred or thousand individuals, such as *Melipona* stingless bees, can rival the diet breadth of colonies housing tens of thousands of individuals, such as *Scaptotrigona*, *Trigona*, or even *Apis mellifera* honeybees. Nevertheless, it is important to note that our findings are derived from studies published until now and are subject to certain limitations. For example, the sampling of larger colonies might not have been sufficiently comprehensive to determine their full diet, given the challenges researchers face in accurately estimating colony sizes. Consequently, it is advisable to interpret our findings with caution.

It has been proposed that larger colonies of corbiculate bees exhibit a higher level of complexity due to the presence of behavioral and morphological castes (Rodriguez-Serrano et al. 2012). In line with these attributes, we suggest that eusocial bees, regardless of colony size, leverage their multiple foragers and sophisticated communication mechanisms (Nieh 2004, Dornhaus and Chittka 2005, Dornhaus et al. 2006, Minahan and Brunet 2020) to efficiently locate and exploit the best available pollen sources in their environment. Once a valuable resource is discovered, these bees are capable of recruiting numerous nestmates to aid in its harvest.

Overall, honey bees (genus *Apis*), bumble bees (genus *Bombus*), and stingless bees (various genera) exhibit unique eusocial strategies and adaptations. For instance, honey bees are characterized by large colonies and advanced foraging mechanisms driven by intricate communication systems, while bumble bees typically have smaller, and in tropical regions, mostly perennial colonies and employ less complex foraging communication methods. In contrast, stingless bees may employ a variety (though less complex than *Apis*) of foraging strategies to collect a diverse range of pollen. These distinctions underscore how each bee group contributes to the development of a more generalized pollen diet within eusocial bee clades, as opposed to solitary bees.

The categorization of bees as specialists or generalists in terms of their pollen diet traditionally referred to their reliance on pollen from a few related plant genera (oligolecty = taxonomic pollen specialist or congeneric flowers) or from plants belonging to multiple families (polylecty) (Wcislo and Cane 1996, Cane and Sipes 2006). However, recent research has challenged this lexical interpretation, as it has been discovered that bees, particularly their larvae, also consume microbes (Steffan et al. 2019). Pollen is rich in various biologically active substances, including proteins, essential amino acids, carbohydrates, lipids, nucleic acids, and phenolic compounds, making it a valuable resource for bees (Komosinska-Vassev et al. 2015, Vaudo et al. 2020). However, microbes, including fungi and bacteria, appear to facilitate the digestion of pollen by promoting fermentation processes within the pollen mass (Steffan et al. 2019). Notably, bee larvae actively consume fungal mycelia during their development (Menezes et al. 2015). Consequently, the term “pollenivory” should be expanded to encompass the omnivorous nature of bees, considering their consumption of both pollen and microbial components (Steffan et al. 2019).

In our study, we focused on measuring the dietary diversity of bees based on the variety of pollen they collect, as quantified by the Shannon–Wiener index, utilizing secondary data. However, it is important to acknowledge that this analysis provides only a partial depiction of their overall diet, considering the wide range of other food sources that bees can potentially consume or encounter. Therefore, a comprehensive examination of the entire diet breadth available and accessible to bees warrants further investigation.

Eusocial bees exhibit a higher level of diversity (*H*ʹ) compared to specialist bees, likely indicating a greater resilience to environmental disturbances. Unlike specialist bees that heavily rely on a narrow range of plant taxa, eusocial bees have a more diverse diet, which reduces their vulnerability to fluctuations in resource availability. Moreover, eusocial bee species possess advantageous traits that enhance their competitive abilities in acquiring and storing food, especially during unfavorable conditions (Michener 1969, 2007). The perennial nature of eusocial colonies, with overlapping generations throughout the year, further contributes to their adaptive advantage over bees in less cooperative societies or solitary lifestyles, which typically have one or a few generations per year (Michener 1969, 2007, Shell and Rehan 2018).

Conversely, the low diversity observed in noneusocial bees suggests that they may have a diminished capacity to cope with adverse scenarios. Given the rapid global deforestation and environmental changes (Sandker et al. 2017), noneusocial bees are particularly susceptible to intense ecological pressures, including food shortages, nutritional deficiencies, and competition with generalist and cooperative eusocial bee lineages (Wcislo and Cane 1996, Müller et al. 2006, Potts et al. 2010). While our study focused on specific attributes when selecting bee species for analysis, it is important to acknowledge that the diet breadth of bees could be further investigated in relation to other characteristics such as body size and lifespan. However, a comprehensive published compilation of all biological features of the bee taxa currently studied is currently lacking. Therefore, we recommend that future palynological analyses incorporate as many relevant characteristics as possible for all bee taxa, facilitating more comprehensive investigations similar to the approach employed in this study. This would greatly enhance our understanding of the intricate relationships between bee biology, diet breadth, and ecological dynamics.

This research, while providing valuable insights, had some inherent limitations. For instance, the overrepresentation of Brazilian studies in stingless bee pollen analysis may have been influenced by the fact that Brazilian researchers have a long-standing history of palynological research, contributing to the accumulation of a substantial amount of data on stingless bees from that country. Furthermore, it is noteworthy that, in the case of obligatory social bee species, it was necessary to consider not only the trophic diversity found in pollen grains but also the population size of the colonies to enable the cross-examination of trophic diversity with colony size.

Finally, we selected the Shannon diversity index over the Simpson index for palynological studies due to its widespread usage in pollen analyses. However, this preference may bias the results, particularly for the stingless bee group. This bias could potentially be mitigated through future research efforts that incorporate pollen analysis for a broader range of species, including those from the *Apis* and *Bombus* genera, as well as solitary bees.

## Conclusions

Our study highlights the critical role of diet breadth in shaping the lifestyle and ecological resilience of bees in the face of ongoing environmental changes. We observed that most bee species are specialists, relying on a narrow range of plant taxa for their survival. However, these specialist bees are increasingly vulnerable to anthropogenic disturbances, including habitat degradation, deforestation, urbanization, agrochemical use, and climate change.

On the other hand, our findings reveal that bees with a more generalist and structured societal organization (eusocial bees) have a broader diet, which may enhance their competitive advantage and resilience in the face of adverse environmental conditions. Importantly, this resilience is not solely dependent on colony size, as even smaller eusocial colonies can exhibit a diverse diet breadth. Therefore, given the increasing threats to bee populations and the potential decline of specialist species, the conservation of these vulnerable bee lineages becomes a challenging task. Efforts should be directed towards understanding and mitigating the impacts of anthropogenic disturbances on specialized bees and promoting habitat preservation, sustainable agricultural practices, and the protection of pollinator-friendly landscapes. Ultimately, addressing the conservation needs of bees, especially those with more restrictive lifestyles, is of utmost importance to safeguard the vital ecosystem services they provide and to ensure the long-term sustainability of our global ecosystems. Thus, to support bees, conservation efforts should primarily concentrate on habitat restoration, pesticide reduction, raising public awareness, and promoting sustainable agriculture, among other strategies (Stout and Dicks 2022, Bergamo et al. 2023).

## Supplementary Material

ieae023_suppl_Supplementary_Appendixs_S1

## References

[CIT0001] Andrade-Silva ACR , Nascimento FS. Multifemale nests and social behavior in *Euglossa melanotricha* (Hymenoptera, Apidae, Euglossini). J Hymenopt Res. 2012:26:1.

[CIT0002] Bergamo PJ , Rito KF, Viana BF, Garcia E, Lughadha EN, Maués MM, Rech AR, Silva FDS, Varassin IG, Agostini K, et al. Integrating public engagement to intensify pollination services through ecological restoration. iScience. 2023:26(8):107276. 10.1016/j.isci.2023.107276 37559905 PMC10407755

[CIT0003] Boff S , Forfert N, Paxton RJ, Montejo E, Quezada-Euan JJG. A behavioral guard caste in a primitively eusocial orchid bee, *Euglossa viridissima*, helps defend the nest against resin theft by conspecifics. Insectes Soc. 2015:62(2):247–249. 10.1007/s00040-015-0397-3

[CIT0004] Bossert S , Murray EA, Almeida EAB, Brady SG, Blaimer BB, Danforth BN. Combining transcriptomes and ultraconserved elements to illuminate the phylogeny of Apidae. Mol Phylogenet Evol. 2019:130:121–131. 10.1016/j.ympev.2018.10.012 30326287

[CIT0005] Branstetter MG , Danforth BN, Pitts JP, Gates MW, Kula RR, Brady G. Phylogenomic insights into the evolution of stinging wasps and the origins of ants and bees. Curr Biol. 2017:27:1019–1025. 28376325 10.1016/j.cub.2017.03.027

[CIT0006] Cane JH , Sipes SS. Characterizing floral specialization by bees: analytical methods and a revised lexicon for oligolecty. In: Waser NM, Ollerton J, editors. Plant–pollinator interactions: from specialization to generalization. Chicago (IL) and London (UK): The University of Chicago Press; 2006. p. 99–122.

[CIT0007] Cardinal S , Danforth BN. The antiquity and evolutionary history of social behavior in bees. PLoS One. 2011:6(6):e21086. 10.1371/journal.pone.0021086 21695157 PMC3113908

[CIT0008] Cardinal S , Danforth B. Bees diversified in the age of eudicots. Proc R Soc B Biol Sci. 2013:280:20122686. 10.1098/rspb.2012.2686 PMC3574388 23363629

[CIT0009] Costa JT , Fitzgerald TD. Social terminology revisited: where are we ten years later? Ann Zool Fennici. 2005:42:559–564.

[CIT0010] Crespi BJ , Yanega D. The definition of eusociality. Behav Ecol. 1995:6(1):109–115. 10.1093/beheco/6.1.109

[CIT0011] Danforth BN , Cardinal S, Praz C, Almeida EAB, Michez D. The impact of molecular data on our understanding of bee phylogeny and evolution. Annu Rev Entomol. 2013:58:57–78. 10.1146/annurev-ento-120811-153633 22934982

[CIT0012] Davison PJ , Field J. Environmental barriers to sociality in an obligate eusocial sweat bee. Insectes Soc. 2018:65(4):549–559. 10.1007/s00040-018-0642-7 30416204 PMC6208632

[CIT0013] Dornhaus A , Chittka L. Bumble bees (*Bombus terrestris*) store both food and information in honeypots. Behav Ecol. 2005:16(3):661–666. 10.1093/beheco/ari040

[CIT0014] Dornhaus A , Klügl F, Oechslein C, Puppe F, Chittka L. Benefits of recruitment in honey bees: effects of ecology and colony size in an individual-based model. Behav Ecol. 2006:17(3):336–344. 10.1093/beheco/arj036

[CIT0015] Felsenstein J. Phylogenies and the comparative method. Am Nat. 1985:125(1):1–15. 10.1086/284325 31094602

[CIT0016] Garland T , Díaz-Uriarte R. Polytomies and phylogenetically independent contrasts: examination of the bounded degrees of freedom approach. Syst Biol. 1999:48(3):547–558. 10.1080/106351599260139 12066293

[CIT0017] Grafen A. The phylogenetic regression. Philos Trans R Soc London Ser B. 1989:326(1233):119–157. 10.1098/rstb.1989.0106 2575770

[CIT0018] Harvey PH , Pagel M. The comparative method in evolutionary biology. Oxford (UK): Oxford University Press; 1991.

[CIT0019] Hines HM. Historical biogeography, divergence times, and diversification patterns of bumble bees (Hymenoptera: Apidae: *Bombus*). Syst Biol. 2008:57(1):58–75. 10.1080/10635150801898912 18275002

[CIT0020] Ihaka R , Gentleman R. R: a language for data analysis and graphics. J Comput Graph Stat. 1996:5(3):299–314. 10.2307/1390807

[CIT0021] Kamilar JM , Cooper N. Phylogenetic signal in primate behaviour, ecology and life history. Philos Trans R Soc London Ser B. 2013:368(1618):20120341. 10.1098/rstb.2012.0341 23569289 PMC3638444

[CIT0022] Komosinska-Vassev K , Olczyk P, Kafmierczak J, Mencner L, Olczyk K. Bee pollen: chemical composition and therapeutic application. Evid Based Complement Alternat Med. 2015:2015:1–16. 10.1155/2015/297425 PMC4377380 25861358

[CIT0023] Magurran AE. Ecological diversity and its measurement. Princeton (NJ): Princeton University Press; 1988

[CIT0024] Martins AC , Melo GAR. The New World oil-collecting bees *Centris* and *Epicharis* (Hymenoptera, Apidae): molecular phylogeny and biogeographic history. Zool Scrip. 2016:45:22–33.

[CIT0025] Martins AC , Melo GAR, Renner SS. The corbiculate bees arose from New World oil-collecting bees: implications for the origin of pollen baskets. Mol Phylogenet Evol. 2014:80:88–94. 10.1016/j.ympev.2014.07.003 25034728

[CIT0026] Menezes C , Vollet-Neto A, Marsaioli AJ, Zampieri D, Fontoura IC, Luchessi AD, Imperatriz-Fonseca VL. A Brazilian social bee must cultivate fungus to survive. Curr Biol. 2015:25(21):2851–2855. 10.1016/j.cub.2015.09.028 26592344

[CIT0027] Michener CD. Comparative social behavior of bees. Annu Rev Entomol. 1969:14(1):299–342. 10.1146/annurev.en.14.010169.001503

[CIT0028] Michener CD. The bees of the word. 2nd ed. Baltimore (MD): Johns Hopkins University Press; 2007.

[CIT0029] Minahan D , Brunet J. Foraging strategy predicts species-specific patterns of pollen foraging by honey bees and bumble bees. Authorea. 2020:6:1–18.

[CIT0030] Müller A , Diener S, Schnyder S, Stutz K, Sedivy C, Dorn S. Quantitative pollen requirements of solitary bees: implications for bee conservation and the evolution of bee-flower relationships. Biol Conserv. 2006:130(4):604–615. 10.1016/j.biocon.2006.01.023

[CIT0031] Nieh JC. Recruitment communication in stingless bees (Hymenoptera, Apidae, Meliponini). Apidologie. 2004:35(2):159–182. 10.1051/apido:2004007

[CIT0032] Omland KE. The assumptions and challenges of ancestral state reconstructions. Syst Biol. 1999:48(3):604–611. 10.1080/106351599260175

[CIT0033] Pagel M. Detecting correlated evolution on phylogenies: a general method for the comparative analysis of discrete characters. Proc R Soc B Biol Sci. 1994:255:37–45.

[CIT0034] Paradis E , Schliep K. ape 5.0: an environment for modern phylogenetics and evolutionary analyses in R. Bioinformatics. 2019:35(3):526–528. 10.1093/bioinformatics/bty633 30016406

[CIT0035] Peters RS , Krogmann L, Mayer C, Donath A, Gunkel S, Meusemann K, Kozlov A, Podsiadlowski L, Petersen M, Lanfear R, et al. Evolutionary history of the Hymenoptera. Curr Biol. 2017:27(7):1013–1018. 10.1016/j.cub.2017.01.027 28343967

[CIT0055] Pinheiro J , Bates D, R Core Team. nlme: Linear and nonlinear mixed effects models. 2020. https://CRAN.R-project.org/package=nlme

[CIT0036] Potts SG , Biesmeijer JC, Kremen C, Neumann P, Schweiger O, Kunin WE. Global pollinator declines: trends, impacts and drivers. Trends Ecol Evol. 2010:25(6):345–353. 10.1016/j.tree.2010.01.007 20188434

[CIT0037] R Core Team. R: a language and environment for statistical computing. Vienna (Austria): The R Foundation for Statistical Computing; 2021.

[CIT0038] Ramírez SR , Roubik DW, Skov C, Pierce NE. Phylogeny, diversification patterns and historical biogeography of euglossine orchid bees (Hymenoptera: Apidae). Biol J Linn Soc. 2010:100(3):552–572. 10.1111/j.1095-8312.2010.01440.x

[CIT0039] Rasmussen C , Cameron SA. Global stingless bee phylogeny supports ancient divergence, vicariance, and long distance dispersal. Biol J Linn Soc. 2010:99(1):206–232. 10.1111/j.1095-8312.2009.01341.x

[CIT0040] Revell LJ. phytools: an R package for phylogenetic comparative biology (and other things). Methods Ecol Evol. 2012:3(2):217–223. 10.1111/j.2041-210x.2011.00169.x

[CIT0041] Revell LJ. Ancestral character estimation under the threshold model from quantitative genetics. Evolution. 2014:68(3):743–759. 10.1111/evo.12300 24152239

[CIT0042] Rodriguez-Serrano E , Inostroza-Michael O, Avaria-Llautureo J, Hernandez CE. Colony size evolution and the origin of eusociality in corbiculate bees (Hymenoptera: Apinae). PLoS One. 2012:7(7):e40838. 10.1371/journal.pone.0040838 22808274 PMC3396608

[CIT0043] Sandker M , Finegold Y, Lindquist E. Global deforestation patterns: comparing recent and past forest loss processes through a spatially explicit analysis. Int For Rev. 2017:19:350–368.

[CIT0044] Shannon CE , Weaver W. The mathematical theory of communication. Urbana (IL): University of Illinois Press; 1949.

[CIT0045] Shell WA , Rehan SM. Behavioral and genetic mechanisms of social evolution: insights from incipiently and facultatively social bees. Apidologie. 2018:43:13–30.

[CIT0046] Soro A , Ayasse M, Zobel MU, Paxton RJ. Kin discriminators in the eusocial sweat bee *Lasioglossum malachurum*: the reliability of cuticular and Dufour’s gland odours. Behav Ecol Sociobiol. 2010:65(4):641–653. 10.1007/s00265-010-1066-1

[CIT0047] Spellerberg IF , Fedor PJ. A tribute to Claude Shannon (1916–2001) and a plea for more rigorous use of species richness, species diversity and the ‘Shannon–Wiener’ Index. Glob Ecol Biogeogr. 2003:12(3):177–179. 10.1046/j.1466-822x.2003.00015.x

[CIT0048] Steffan SA , Dharampal PS, Danforth BN, Gaines-Day HR, Takizawa Y, Chikaraishi Y. Omnivory in bees: elevated trophic positions among all major bee families. Am Nat. 2019:194(3):414–421. 10.1086/704281 31553217

[CIT0049] Stout JC , Dicks LV. From science to society: implementing effective strategies to improve wild pollinator health. Philos Transac R Soc. 2022:377(1853):20210165. 10.1098/rstb.2021.0165 PMC9058532 35491595

[CIT0050] Vaudo AD , Tooker JF, Patch HM, Biddinger DJ, Coccia M, Crone MK, Fiely M, Francis JS, Hines HM, Hodges M, et al. Pollen protein: lipid macronutrient ratios may guide broad patterns of bee species floral preferences. Insects. 2020:11(2):132. 10.3390/insects11020132 32085627 PMC7074338

[CIT0051] Vit P , Pedro SRM, Roubik DW. Pot-pollen in stingless bee melittology. Cham (Switzerland): Springer International Publishing; 2018.

[CIT0052] Wcislo WT , Cane JH. Floral resource utilization by solitary bees (Hymenoptera: Apoidea) and exploitation of their stored foods by natural enemies. Annu Rev Entomol. 1996:41:257–286. 10.1146/annurev.en.41.010196.001353 15012330

[CIT0053] Wilson EO. The insect societies. Cambridge (MA, USA): The Belknap Press of Harvard University; 1971.

[CIT0054] Wilson EO , Holldobler B. Eusociality: origin and consequences. Proc Natl Acad Sci USA. 2005:102(38):13367–13371. 10.1073/pnas.0505858102 16157878 PMC1224642

