# Supplementary material for: Pollen diet diversity across bee lineages varies with lifestyle rather than colony size
Source: J Insect Sci. 2024 Mar 5;24(2):1. doi: 10.1093/jisesa/ieae023 (PMC10914374; doi:10.1093/jisesa/ieae023)
Supplement: ieae023_suppl_Supplementary_Appendixs_S1 [file ieae023_suppl_supplementary_appendixs_s1.pdf]

DIET DIVERSITY

Lifestyle	Species	Diet breadth	Study Site	References
Eusocial	*Apis cerana*	0.65	China (Mengzi)	2013 Liu etal (Sociobiology)
Eusocial	*Apis mellifera*	0.01	USA (Wisconsin)	2019 Guzman (agriculture, Ecosystems and Environment)
Eusocial	*Apis mellifera*	0.25	Spain (Canary Islands - Tenerife)	2019 Barroso-Arévalo (Spanish Journal of Agricultural Research)
Eusocial	*Apis mellifera*	0.57	China (Mengzi)	2013 Liu etal (Sociobiology)
Eusocial	*Apis mellifera*	1.45	Uganda (Bwindi Impenetrable National Park)	2006 Kajobe (African.J.Ecol.)
Eusocial	*Apis mellifera*	1.6	USA (Wisconsin)	2019 Guzman (Agriculture, Ecosystems and Environment)
Eusocial	*Apis mellifera*	2.23	USA (Tennessee)	2018 Alburaki (bioone)
Eusocial	*Apis mellifera*	2.26	USA (Tennessee)	2018 Alburaki (bioone)
Eusocial	*Apis mellifera*	2.35	USA (Tennessee)	2018 Alburaki (bioone)
Eusocial	*Apis mellifera*	2.43	USA (Tennessee)	2018 Alburaki (bioone)
Eusocial	*Apis mellifera*	2.66	Brazil (Mucugê - BA)	2009 Franco etal (Sociobiology)
Eusocial	*Apis mellifera*	2.71	Brazil (Itatim - BA)	2003 Aguiar etal (Rev.Bra.Zoo.)
Eusocial	*Apis mellifera*	2.78	Spain (Canary Islands - Tenerife)	2019 Barroso-Arévalo (Spanish Journal of Agricultural Research)
Eusocial	*Apis mellifera*	3.17	Brazil (Piracicaba - SP)	1999 Carvalho etal (Bragantia)
Eusocial	*Apis mellifera*	3.17	Brazil (Piracicaba - SP)	2003 Oliveira (Dissertação)
Eusocial	*Apis mellifera*	4.4	Brazil (Serra do Mar - SP)	1996 Wilms etal (Stud.Neotrp.Fau.Envir.)
Eusocial	*Apotrigona nebulata*	0.64	Uganda (Bwindi Impenetrable National Park)	2006 Kajobe (African.J.Ecol.)
Eusocial	*Bombus morio*	0.46	Brazil (Ribeirão Preto - SP)	1989 Camilo & Garófalo (J.Tropical.Ecol.)
Eusocial	*Bombus morio*	1.86	Brazil (Ribeirão Preto - SP)	1989 Camilo & Garófalo (J.Tropical.Ecol.)
Eusocial	*Bombus morio*	1.87	Brazil (Ribeirão Preto - SP)	1989 Camilo & Garófalo (J.Tropical.Ecol.)
Eusocial	*Bombus pauloensis*	0.39	Brazil (Ribeirão Preto - SP)	1989 Camilo & Garófalo (J.Tropical.Ecol.)
Eusocial	*Bombus pauloensis*	1.96	Brazil (Ribeirão Preto - SP)	1989 Camilo & Garófalo (J.Tropical.Ecol.)
Eusocial	*Bombus pauloensis*	1.99	Brazil (Ribeirão Preto - SP)	1989 Camilo & Garófalo (J.Tropical.Ecol.)
Eusocial	*Bombus pauloensis*	2.38	Brazil (Mucugê - BA)	2009 Franco etal (Sociobiology)
Eusocial	*Bombus terrestris*	0.86	UK (Hampshire, Dorset or Wiltshire)	2002 Goulson (Oecologia)
Eusocial	*Cephalotrigona femorata*	2.34	Brazil (Manaus - AM)	2009 Oliveira (Acta.Amazonica)
Eusocial	*Eomelipona bicolor*	3.8	Brazil (Serra do Mar - SP)	1996 Wilms etal (Stud.Neotrp.Fau.Envir.)
Eusocial	*Frieseomelitta doederleini*	2.07	Brazil (São João do Cariri - PB)	2013 Aguiar etal (Apidologie)
Eusocial	*Frieseomelitta silvestrii*	0.27	Brazil (Belo Horizonte - MG)	2013 Antonini et al. (Urban Eco.)
Eusocial	*Frieseomelitta silvestrii*	1.14	Brazil (Itatim - BA)	2003 Aguiar etal (Rev.Bra.Zoo.)
Eusocial	*Frieseomelitta varia*	1.02	Brazil (Manaus - AM)	2021 Ferreira (J. Apic. Res.)
Eusocial	*Frieseomelitta varia*	2.04	Brazil (Manaus - AM)	2021 Ferreira (J. Apic. Res.)
Eusocial	*Frieseomelitta varia*	3.11	Brazil (Manaus - AM)	2003 Oliveira (Dissertação)
Eusocial	*Geotrigona argentina*	1.38	Argentina (Chaco)	2012 Vossler (Thesis)
Eusocial	*Geotrigona mombuca*	1.94	Brazil (Belo Horizonte - MG)	2013 Antonini et al. (Urban Eco.) 1960 Lindauer and Kerr
Eusocial	*Geotrigona subterranea*	0.48	Brazil (Belo Horizonte - MG)	2013 Antonini et al. (Urban Eco.)
Eusocial	*Geotrigona subterranea*	0.92	Brazil (Belo Horizonte - MG)	2013 Antonini et al. (Urban Eco.)
Eusocial	*Geotrigona subterranea*	1.58	Brazil (Belo Horizonte - MG)	2013 Antonini et al. (Urban Eco.)
Eusocial	*Melikerria fasciculata*	3.43	Brazil (Bequimão - MA)	2018 Gostinski (Dissertação)
Eusocial	*Melipona (Melikerria) beecheii*	0.86	Costa Rica (El Sur de Turrubares)	1999 Biesmeijer etal (Apidologie)
Eusocial	*Melipona (Melikerria) beecheii*	1.46	Costa Rica (El Sur de Turrubares)	1999 Biesmeijer etal (Apidologie)
Eusocial	*Melipona (Michmelia) fulva*	2.38	Brazil (Manaus - AM)	2009 Oliveira (Acta.Amazonica)
Eusocial	*Melipona interrupta*	0.07	Brazil (Manaus - AM)	2015 Ferreira & Absy (Arthropod-Plant Interactions)
Eusocial	*Melipona interrupta*	0.13	Brazil (Manaus - AM)	2021 Ferreira (J. Apic. Res.)
Eusocial	*Melipona interrupta*	1.4	Brazil (Manaus - AM)	2021 Ferreira (J. Apic. Res.)

Eusocial	*Melipona interrupta*	1.53	Brazil (Manaus - AM)	2015 Ferreira & Absy (Arthropod-Plant Interactions)
Eusocial	*Melipona marginata*	3.3	Brazil (Serra do Mar - SP)	1996 Wilms etal (Stud.Neotrp.Fau.Envir.)
Eusocial	*Melipona quadrifasciata*	0.4	Brazil (Belo Horizonte - MG)	2013 Antonini et al. (Urban Eco.)
Eusocial	*Melipona quadrifasciata*	0.82	Brazil (Ribeirão Preto - SP)	2014 Maia-Silva etal (Sociobiology)
Eusocial	*Melipona quadrifasciata*	0.88	Brazil (Belo Horizonte - MG)	2013 Antonini et al. (Urban Eco.)
Eusocial	*Melipona quadrifasciata*	1.47	Brazil (Belo Horizonte - MG)	2013 Antonini et al. (Urban Eco.)
Eusocial	*Melipona quadrifasciata*	2.09	Brazil (Ribeirão Preto - SP)	2014 Maia-Silva etal (Sociobiology)
Eusocial	*Melipona quadrifasciata*	3.18	Brazil (Serra do Mar - SP)	1996 Wilms etal (Stud.Neotrp.Fau.Envir.)
Eusocial	*Melipona quadrifasciata anthidioides*	1.25	Brazil (Chapada Diamantina - BA)	2020 Machado (Landscape Ecol.)
Eusocial	*Melipona seminigra*	2.57	Brazil (Manaus - AM)	2009 Oliveira (Acta.Amazonica)
Eusocial	*Melipona seminigra*	2.97	Brazil (Manaus - AM)	2009 Oliveira (Acta.Amazonica)
Eusocial	*Melipona seminigra merrillae*	0.34	Brazil (Manaus - AM)	2015 Ferreira & Absy (Arthropod-Plant Interactions)
Eusocial	*Melipona seminigra merrillae*	0.46	Brazil (Manaus - AM)	2021 Ferreira (J. Apic. Res.)
Eusocial	*Melipona seminigra merrillae*	1.46	Brazil (Manaus - AM)	2021 Ferreira (J. Apic. Res.)
Eusocial	*Melipona seminigra merrillae*	1.74	Brazil (Manaus - AM)	2015 Ferreira & Absy (Arthropod-Plant Interactions)
Eusocial	*Melipona subnitida*	1.01	Brazil (Ribeirão Preto - SP)	2014 Maia-Silva etal (Sociobiology)
Eusocial	*Melipona subnitida*	1.97	Brazil (Ribeirão Preto - SP)	2014 Maia-Silva etal (Sociobiology)
Eusocial	*Meliponula bocandei*	0.9	Uganda (Bwindi Impenetrable National Park)	2006 Kajobe (African.J.Ecol.)
Eusocial	*Michmelia fasciata*	1.47	Costa Rica (El Sur de Turrubares)	1999 Biesmeijer etal (Apidologie)
Eusocial	*Michmelia fasciata*	1.58	Costa Rica (El Sur de Turrubares)	1999 Biesmeijer etal (Apidologie)
Eusocial	*Michmelia flavolineata*	3.41	Brazil (Bequimão - MA)	2018 Gostinski (Dissertação)
Eusocial	*Michmelia rufiventris*	3.3	Brazil (Serra do Mar - SP)	1996 Wilms etal (Stud.Neotrp.Fau.Envir.)
Eusocial	*Michmelia scutellaris*	2.27	Brazil (Planaltino - BA)	2017 Lucas et al. (Grana)
Eusocial	*Michmelia scutellaris*	2.36	Brazil (Planaltino - BA)	2017 Lucas et al. (Grana)
Eusocial	*Nannotrigona testaceicornis*	0.3	Brazil (Belo Horizonte - MG)	2013 Antonini et al. (Urban Eco.)
Eusocial	*Nannotrigona testaceicornis*	1.28	Brazil (Belo Horizonte - MG)	2013 Antonini et al. (Urban Eco.)
Eusocial	*Nannotrigona testaceicornis*	1.42	Brazil (Belo Horizonte - MG)	2013 Antonini et al. (Urban Eco.)
Eusocial	*Nannotrigona testaceicornis*	1.5	Brazil (Belo Horizonte - MG)	2013 Antonini et al. (Urban Eco.)
Eusocial	*Nannotrigona testaceicornis*	1.95	Brazil (Belo Horizonte - MG)	2013 Antonini et al. (Urban Eco.)
Eusocial	*Nannotrigona testaceicornis*	2.69	Brazil (Piracicaba - SP)	1999 Carvalho etal (Bragantia)
Eusocial	*Paratrigona subnuda*	1.38	Brazil (Belo Horizonte - MG)	2013 Antonini et al. (Urban Eco.)
Eusocial	*Paratrigona subnuda*	1.94	Brazil (Belo Horizonte - MG)	2013 Antonini et al. (Urban Eco.)
Eusocial	*Paratrigona subnuda*	2.48	Brazil (Belo Horizonte - MG)	2013 Antonini et al. (Urban Eco.)
Eusocial	*Paratrigona subnuda*	4.28	Brazil (Serra do Mar - SP)	1996 Wilms etal (Stud.Neotrp.Fau.Envir.)
Eusocial	*Partamona helleri*	0.92	Brazil (Belo Horizonte - MG)	2013 Antonini et al. (Urban Eco.)
Eusocial	*Partamona helleri*	2.95	Brazil (Piracicaba - SP)	1999 Carvalho etal (Bragantia)
Eusocial	*Partamona helleri*	4	Brazil (Serra do Mar - SP)	1996 Wilms etal (Stud.Neotrp.Fau.Envir.)
Eusocial	*Partamona rustica*	1.01	Brazil (Iramaia - BA)	2016 Miranda (thesis)
Eusocial	*Partamona rustica*	1.08	Brazil (Boa Vista do Tupim - BA)	2016 Miranda (thesis)
Eusocial	*Partamona rustica*	1.09	Brazil (Contendas do Sincorá - BA)	2016 Miranda (thesis)
Eusocial	*Partamona rustica*	1.12	Brazil (Ituaçu - BA)	2016 Miranda (thesis)
Eusocial	*Partamona rustica*	1.74	Brazil (Manoel Vitorino - BA)	2016 Miranda (thesis)
Eusocial	*Partamona rustica*	1.83	Brazil (Macaúbas - BA)	2016 Miranda (thesis)
Eusocial	*Partamona rustica*	2.05	Brazil (Cocos - BA)	2016 Miranda (thesis)
Eusocial	*Partamona rustica*	2.17	Brazil (Milagre - BA)	2016 Miranda (thesis)
Eusocial	*Partamona rustica*	2.3	Brazil (Ruy Barbosa - BA)	2016 Miranda (thesis)
Eusocial	*Partamona rustica*	2.41	Brazil (Tanque Novo - BA)	2016 Miranda (thesis)
Eusocial	*Partamona rustica*	2.73	Brazil (Conego Marinho - MG)	2016 Miranda (thesis)

Eusocial	*Plebeia droryana*	1	Brazil (Belo Horizonte - MG)	2013 Antonini et al. (Urban Eco.)
Eusocial	*Plebeia droryana*	1.31	Brazil (Belo Horizonte - MG)	2013 Antonini et al. (Urban Eco.)
Eusocial	*Plebeia droryana*	1.68	Brazil (Belo Horizonte - MG)	2013 Antonini et al. (Urban Eco.)
Eusocial	*Plebeia droryana*	3.16	Brazil (Piracicaba - SP)	1999 Carvalho etal (Bragantia)
Eusocial	*Plebeia droryana*	3.16	Brazil (Piracicaba - SP)	2003 Oliveira (Dissertação)
Eusocial	*Plebeia droryana*	4.3	Brazil (Serra do Mar - SP)	1996 Wilms etal (Stud.Neotrp.Fau.Envir.)
Eusocial	*Plebeia remota*	3.67	Brazil (Serra do Mar - SP)	1996 Wilms etal (Stud.Neotrp.Fau.Envir.)
Eusocial	*Scaptotrigona depilis*	4.01	Brazil (Ribeirão Preto - SP)	2017 Aleixo etal (Apidologie)
Eusocial	*Scaptotrigona mexicana*	1.8	Ref. Não encontrada	2018 Vit (BOOK)
Eusocial	*Scaptotrigona postica*	0.43	Brazil (Belo Horizonte - MG)	2013 Antonini et al. (Urban Eco.) 1960 Lindauer and Kerr
Eusocial	*Schwarziana quadripunctata*	3.84	Brazil (Serra do Mar - SP)	1996 Wilms etal (Stud.Neotrp.Fau.Envir.)
Eusocial	*Tetragona clavipes*	1.96	Brazil (Belo Horizonte - MG)	2013 Antonini et al. (Urban Eco.)
Eusocial	*Tetragonisca angustula*	0.47	Brazil (Belo Horizonte - MG)	2013 Antonini et al. (Urban Eco.)
Eusocial	*Tetragonisca angustula*	0.57	Bolivia (Amboró - Santa Cruz)	2018 Saravia-Navia (ecology, behavior and bionomics)
Eusocial	*Tetragonisca angustula*	0.73	Bolivia (Amboró - Santa Cruz)	2018 Saravia-Navia (ecology, behavior and bionomics)
Eusocial	*Tetragonisca angustula*	1.33	Brazil (Belo Horizonte - MG)	2013 Antonini et al. (Urban Eco.)
Eusocial	*Tetragonisca angustula*	1.33	Brazil (Belo Horizonte - MG)	2013 Antonini et al. (Urban Eco.)
Eusocial	*Tetragonisca angustula*	1.95	Brazil (Belo Horizonte - MG)	2013 Antonini et al. (Urban Eco.)
Eusocial	*Tetragonisca angustula*	2.43	Brazil (Belo Horizonte - MG)	2013 Antonini et al. (Urban Eco.)
Eusocial	*Tetragonisca angustula*	2.7	Brazil (Piracicaba - SP)	1999 Carvalho etal (Bragantia)
Eusocial	*Tetragonisca angustula*	2.7	Brazil (Piracicaba - SP)	1999 Carvalho etal (Bragantia)
Eusocial	*Tetragonisca fiebrigi*	2.24	Argentina (Chaco)	2012 Vossler (Thesis)
Eusocial	*Trigona chanchamayoensis*	2.2	Brazil (Corumbá - MS)	2003 Oliveira (Dissertação)
Eusocial	*Trigona fulviventris*	1.15	Brazil (Belo Horizonte - MG)	2013 Antonini et al. (Urban Eco.)
Eusocial	*Trigona fulviventris*	3.39	Brazil (Manaus - AM)	2009 Oliveira (Acta.Amaz.) 1998 Slaa et al. (J. of Api. Res.) 2014 Jaffé etal (Naturw.)
Eusocial	*Trigona hyalinata*	0.95	Brazil (Belo Horizonte - MG)	2013 Antonini et al. (Urban Eco.)
Eusocial	*Trigona hyalinata*	1.96	Brazil (Belo Horizonte - MG)	2013 Antonini et al. (Urban Eco.)
Eusocial	*Trigona spinipes*	1.11	Brazil (Belo Horizonte - MG)	2013 Antonini et al. (Urban Eco.)
Eusocial	*Trigona spinipes*	1.25	Brazil (Belo Horizonte - MG)	2013 Antonini et al. (Urban Eco.)
Eusocial	*Trigona spinipes*	1.32	Brazil (Belo Horizonte - MG)	2013 Antonini et al. (Urban Eco.)
Eusocial	*Trigona spinipes*	1.85	Brazil (Belo Horizonte - MG)	2013 Antonini et al. (Urban Eco.)
Eusocial	*Trigona spinipes*	1.95	Brazil (Belo Horizonte - MG)	2013 Antonini et al. (Urban Eco.)
Eusocial	*Trigona spinipes*	2.22	Brazil (São João do Cariri - PB)	2013 Aguiar etal (Apidologie)
Eusocial	*Trigona spinipes*	2.31	Brazil (Itatim - BA)	2003 Aguiar etal (Rev.Bra.Zoo.)
Eusocial	*Trigona spinipes*	4.25	Brazil (Serra do Mar - SP)	1996 Wilms etal (Stud.Neotrp.Fau.Envir.)
Eusocial	*Trigona williana*	2.83	Brazil (Manaus - AM)	2003 Oliveira (Dissertação)
Non-eusocial	*Caenonomada unicalcarata*	1.44	Brazil (São João do Cariri - PB)	2013 Aguiar etal (Apidologie)
Non-eusocial	*Ceblurgus longipalpis*	0.15	Brazil (São João do Cariri - PB)	2013 Aguiar etal (Apidologie)
Non-eusocial	*Centris aenea*	1.49	Brazil (Itatim - BA)	2003 Aguiar etal (Rev.Bra.Zoo.)
Non-eusocial	*Centris aenea*	1.56	Brazil (Uberlândia - MG)	2015 Rabelo etal (Apidologie)
Non-eusocial	*Centris analis*	0.36	Brazil (Ribeirão Preto - SP)	2017 Silva etal (Arthropod-Plant Interactions)
Non-eusocial	*Centris analis*	0.88	Brazil (Uberlândia - MG)	2015 Rabelo etal (Apidologie)
Non-eusocial	*Centris analis*	1.83	Brazil (Ribeirão Preto - SP)	2017 Silva etal (Arthropod-Plant Interactions)
Non-eusocial	*Centris burgdorfi*	1.09	Brazil (Ponta Grossa - PR)	2019 Sabino etal (Arthropod-Plant Interactions)
Non-eusocial	*Centris burgdorfi*	1.17	Brazil (Natal - RN)	2019 Sabino etal (Arthropod-Plant Interactions)
Non-eusocial	*Centris burgdorfi*	1.71	Brazil (Cavalcante - GO)	2019 Sabino etal (Arthropod-Plant Interactions)
Non-eusocial	*Centris denudans*	1.19	Brazil (Uberlândia - MG)	2015 Rabelo etal (Apidologie)
Non-eusocial	*Centris flavifrons*	1.95	Brazil (Uberlândia - MG)	2015 Rabelo etal (Apidologie)

Non-eusocial	*Centris longimana*	1.4	Brazil (Uberlândia - MG)	2015 Rabelo etal (Apidologie)
Non-eusocial	*Centris mocsaryi*	0.67	Brazil (Uberlândia - MG)	2015 Rabelo etal (Apidologie)
Non-eusocial	*Centris nitens*	1.33	Brazil (Uberlândia - MG)	2015 Rabelo etal (Apidologie)
Non-eusocial	*Centris poecila*	0.86	Brazil (Uberlândia - MG)	2015 Rabelo etal (Apidologie)
Non-eusocial	*Centris scopipes*	1.28	Brazil (Uberlândia - MG)	2015 Rabelo etal (Apidologie)
Non-eusocial	*Centris spilopoda*	1.1	Brazil (Uberlândia - MG)	2015 Rabelo etal (Apidologie)
Non-eusocial	*Centris tarsata*	0.71	Brzil (Nova Soure - BA)	2015 Cruz etal (Acta.Bot.Brasil.)
Non-eusocial	*Centris varia*	1.4	Brazil (Uberlândia - MG)	2015 Rabelo etal (Apidologie)
Non-eusocial	*Ceratina calcarata*	0.35	USA (Durham - NH)	2016 Lawson etal (Behav.Ecol.Sociobiol.)
Non-eusocial	*Ceratina calcarata*	1.37	USA (Durham - NH)	2016 Lawson etal (Behav.Ecol.Sociobiol.)
Non-eusocial	*Diadasia rinconis*	1.06	USA (Tarrant, Dallas, Hunt, Lamar, Travis, Hays, and Burnet)	2016 Ritchie etal (Environ.Entomol.)
Non-eusocial	*Diadasina riparia*	0.96	Brazil (Itatim - BA)	2003 Aguiar etal (Rev.Bra.Zoo.)
Non-eusocial	*Dialictus opacus*	1.83	Brazil (Itatim - BA)	2003 Aguiar etal (Rev.Bra.Zoo.)
Non-eusocial	*Dialictus opacus*	2.24	Brazil (São João do Cariri - PB)	2013 Aguiar etal (Apidologie)
Non-eusocial	*Epicharis affinis*	1.31	Brazil (Uberlândia - MG)	2015 Rabelo etal (Apidologie)
Non-eusocial	*Epicharis albofasciata*	0.55	Brazil (Uberlândia - MG)	2015 Rabelo etal (Apidologie)
Non-eusocial	*Epicharis bicolor*	1.37	Brazil (Uberlândia - MG)	2015 Rabelo etal (Apidologie)
Non-eusocial	*Epicharis dejeanii*	1.6	Brazil (Cananéia - SP)	2014 Faria (Thesis)
Non-eusocial	*Epicharis dejeanii*	1.69	Brazil (Bertioga - SP)	2014 Faria (Thesis)
Non-eusocial	*Epicharis flava*	1.5	Brazil (Uberlândia - MG)	2015 Rabelo etal (Apidologie)
Non-eusocial	*Euglossa annectans*	0.1	Brazil (Ilha de Santa Catarina - SC)	2009 Cortopassi-Laurino etal (Genetics and Molecular Research)
Non-eusocial	*Euglossa annectans*	0.71	Brazil (Ilha de Santa Catarina - SC)	2009 Cortopassi-Laurino etal (Genetics and Molecular Research)
Non-eusocial	*Euglossa annectans*	1.47	Brazil (Ilha de Santa Catarina - SC)	2009 Cortopassi-Laurino etal (Genetics and Molecular Research)
Non-eusocial	*Euglossa annectans*	1.65	Brazil (Ilha de Santa Catarina - SC)	2009 Cortopassi-Laurino etal (Genetics and Molecular Research)
Non-eusocial	*Euglossa cordata*	0.13	Brazil (São Luís - MA)	2019 Pinto (sociobiology)
Non-eusocial	*Euglossa cordata*	1.37	Brazil (São Luís - MA)	2019 Pinto (sociobiology)
Non-eusocial	*Euglossa cordata*	1.97	Brazil (São Carlos - SP)	2017 Lima (Dissertação)
Non-eusocial	*Eulaema mocsaryi*	1.35	Brazil (Itacoatiara - AM)	2010 Santos & Absy (Neotrop.Entomol.)
Non-eusocial	*Eulaema nigrita*	1.27	Brazil (São Carlos - SP)	2017 Lima (Dissertação)
Non-eusocial	*Exomalopsis analis*	1.22	Brazil (São João do Cariri - PB)	2013 Aguiar etal (Apidologie)
Non-eusocial	*Exomalopsis analis*	1.39	Brazil (Itatim - BA)	2003 Aguiar etal (Rev.Bra.Zoo.)
Non-eusocial	*Megachile maculata*	0.87	Brazil (Ouro Preto - MG)	2016 Sabino etal (J.KansasEntomol.Soc.)
Non-eusocial	*Melissodes tepaneca*	0.79	USA (Tarrant, Dallas, Hunt, Lamar, Travis, Hays, and Burnet)	2016 Ritchie etal (Environ.Entomol.)
Non-eusocial	*Melitomella grisescens*	0.79	Brazil (São João do Cariri - PB)	2013 Aguiar etal (Apidologie)
Non-eusocial	*Pseudaugochlora pandora*	1.97	Brazil (Itatim - BA)	2003 Aguiar etal (Rev.Bra.Zoo.)
Non-eusocial	*Tetrapedia curvitarsis*	1.63	Brazil (Luiz Antônio - SP)	2014 Faria (Thesis)
Non-eusocial	*Tetrapedia diversipes*	2.24	Brazil (Fortaleza - CE)	2016 Cavalcante (Dissertação)
Non-eusocial	*Tetrapedia rugulosa*	0.98	Brazil (Luiz Antônio - SP)	2014 Faria (Thesis)
Non-eusocial	*Xylocopa frontalis*	0.6	Brazil (Itacoatiara - AM)	2010 Santos & Absy (Neotrop.Entomol.)
Non-eusocial	*Xylocopa grisescens*	1.4	Brazil (Itatim - BA)	2003 Aguiar etal (Rev.Bra.Zoo.)

COLONY SIZE

Species	Colony Size	Study Site	References
Apis cerana	34000	Indonesia (Sumatra)	2013 Koetz (Insects)
Apis mellifera	50000	Thailand	2012 Suwannapong etal (book chapter) ; 2013 Koetz (Insects)
Apis mellifera	50000	Thailand	2012 Suwannapong etal (book chapter) ; 2013 Koetz (Insects)
Apis mellifera	50000	Thailand	2012 Suwannapong etal (book chapter) ; 2013 Koetz (Insects)
Apis mellifera	50000	Thailand	2012 Suwannapong etal (book chapter) ; 2013 Koetz (Insects)
Apis mellifera	50000	Thailand	2012 Suwannapong etal (book chapter) ; 2013 Koetz (Insects)
Apis mellifera	50000	Thailand	2012 Suwannapong etal (book chapter) ; 2013 Koetz (Insects)
Apis mellifera	50000	Thailand	2012 Suwannapong etal (book chapter) ; 2013 Koetz (Insects)
Apis mellifera	50000	Thailand	2012 Suwannapong etal (book chapter) ; 2013 Koetz (Insects)
Apis mellifera	50000	Thailand	2012 Suwannapong etal (book chapter) ; 2013 Koetz (Insects)
Apis mellifera	50000	Thailand	2012 Suwannapong etal (book chapter) ; 2013 Koetz (Insects)
Apis mellifera	50000	Thailand	2012 Suwannapong etal (book chapter) ; 2013 Koetz (Insects)
Apis mellifera	50000	Thailand	2012 Suwannapong etal (book chapter) ; 2013 Koetz (Insects)
Apis mellifera	50000	Thailand	2012 Suwannapong etal (book chapter) ; 2013 Koetz (Insects)
Apis mellifera	50000	Thailand	2012 Suwannapong etal (book chapter) ; 2013 Koetz (Insects)
Apis mellifera	50000	Thailand	2012 Suwannapong etal (book chapter) ; 2013 Koetz (Insects)
Apotrigona nebulata	1800	Uganda (Bwindi Impenetrable National Park)	2006 Kajobe (African.J.Ecol.)
Bombus morio	67	Brazil (Alexandra - PR)	2015 del Castilho etal (Ecol.Evol.)
Bombus morio	67	Brazil (Alexandra - PR)	2015 del Castilho etal (Ecol.Evol.)
Bombus morio	67	Brazil (Alexandra - PR)	2015 del Castilho etal (Ecol.Evol.)
Bombus pauloensis	54	Colombia (Facatativa); Ecuador (Loja)	2015 del Castilho etal (Ecol.Evol.)
Bombus pauloensis	54	Colombia (Facatativa); Ecuador (Loja)	2015 del Castilho etal (Ecol.Evol.)
Bombus pauloensis	54	Colombia (Facatativa); Ecuador (Loja)	2015 del Castilho etal (Ecol.Evol.)
Bombus pauloensis	54	Colombia (Facatativa); Ecuador (Loja)	2015 del Castilho etal (Ecol.Evol.)
Bombus terrestris	150	New Zealand (Hobart)	2015 del Castilho etal (Ecol.Evol.)
Cephalotrigona femorata	2000	NA	Per.Comm. Anderson Fernandes Miranda; Denilce Lopes
Eomelipona bicolor	425	Average colony size	2004 Tóth et al. (Insect. Soc)
Frieseomelitta doederleini	1000	Brazil (São João do Carri - PA)	2013 Aguiar etal (Apidologie)
Frieseomelitta silvestrii	600	Brazil (Itatim - BA)	2003 Aguiar etal (Rev Bra. Zoo.)
Frieseomelitta silvestrii	600	Brazil (Itatim - BA)	2003 Aguiar etal (Rev Bra. Zoo.)
Frieseomelitta varia	1200	Average colony size	2004 Tóth et al. (Insect. Soc)
Frieseomelitta varia	1200	Average colony size	2004 Tóth et al. (Insect. Soc)
Frieseomelitta varia	1200	Average colony size	2004 Tóth et al. (Insect. Soc)
Geotrigona argentina	2500	NA	NA
Geotrigona mombuca	1500	Average colony size	2004 Tóth et al. (Insect. Soc)
Geotrigona subterranea	7485	Brazil (Lontra and Januária - MG)	Barbosa etal (2013)
Geotrigona subterranea	7485	Brazil (Lontra and Januária - MG)	Barbosa etal (2013)
Geotrigona subterranea	7485	Brazil (Lontra and Januária - MG)	Barbosa etal (2013)
Melikerria fasciculata	776	NA	2001 Kerr etal (Rev.Bras.Zool.)
Melipona interrupta	400	Brazil (Manaus - AM)	2015 Ferreira & Absy (Arthropod-Plant Interactions)
Melipona interrupta	400	Brazil (Manaus - AM)	2015 Ferreira & Absy (Arthropod-Plant Interactions)
Melipona interrupta	400	Brazil (Manaus - AM)	2015 Ferreira & Absy (Arthropod-Plant Interactions)
Melipona interrupta	400	Brazil (Manaus - AM)	2015 Ferreira & Absy (Arthropod-Plant Interactions)
Melipona (Melikerria) beecheii	1192	Average colony size	2004 Tóth et al. (Insect. Soc)
Melipona (Melikerria) beecheii	1192	Average colony size	2004 Tóth et al. (Insect. Soc)

Melipona (Michmelia) fulva	539	French Guiana	1979 Roubik (J.Kansas.Entomol.Soc.)
Melipona seminigra	400	Brazil (Manaus - AM)	2009 Oliveira (Acta.Amaz.) ; 2015 Ferreira & Absy (Arthropod-Plant Interactions)
Melipona seminigra	400	Brazil (Manaus - AM)	2009 Oliveira (Acta.Amaz.) ; 2015 Ferreira & Absy (Arthropod-Plant Interactions)
Melipona seminigra merrillae	400	Brazil (Manaus - AM)	2009 Oliveira (Acta.Amaz.) ; 2015 Ferreira & Absy (Arthropod-Plant Interactions)
Melipona seminigra merrillae	400	Brazil (Manaus - AM)	2009 Oliveira (Acta.Amaz.) ; 2015 Ferreira & Absy (Arthropod-Plant Interactions)
Melipona seminigra merrillae	400	Brazil (Manaus - AM)	2009 Oliveira (Acta.Amaz.) ; 2015 Ferreira & Absy (Arthropod-Plant Interactions)
Melipona seminigra merrillae	400	Brazil (Manaus - AM)	2009 Oliveira (Acta.Amaz.) ; 2015 Ferreira & Absy (Arthropod-Plant Interactions)
Melipona marginata	1330	Average colony size	2004 Tóth et al. (Insect. Soc)
Melipona quadrifasciata anthidioides	900	Average colony size	2004 Tóth et al. (Insect. Soc)
Melipona quadrifasciata	900	Average colony size	2004 Tóth et al. (Insect. Soc)
Melipona quadrifasciata	900	Average colony size	2004 Tóth et al. (Insect. Soc)
Melipona quadrifasciata	900	Average colony size	2004 Tóth et al. (Insect. Soc)
Melipona quadrifasciata	900	Average colony size	2004 Tóth et al. (Insect. Soc)
Melipona quadrifasciata	900	Average colony size	2004 Tóth et al. (Insect. Soc)
Melipona quadrifasciata	900	Average colony size	2004 Tóth et al. (Insect. Soc)
Melipona subnitida	700	Average colony size	2004 Tóth et al. (Insect. Soc)
Melipona subnitida	700	Average colony size	2004 Tóth et al. (Insect. Soc)
Meliponula bocandei	1300	Uganda (Bwindi Impenetrable National Park)	2006 Kajobe (African.J.Ecol.)
Michmelia fasciata	1128	Average colony size	2004 Tóth et al. (Insect. Soc)
Michmelia fasciata	1128	Average colony size	2004 Tóth et al. (Insect. Soc)
Michmelia flavolineata	1000	Brazil (Xapuri - AC)	2009 Hilário & Imperatriz-Fonseca (Gene.Mol.Res.)
Michmelia rufiventris	1000	Brazil (Ribeirão Preto - SP)	1974 Camilo-Atique (MSc. Dissertation)
Michmelia scutellaris	1500	Average colony size	2004 Tóth et al. (Insect. Soc)
Michmelia scutellaris	1500	Average colony size	2004 Tóth et al. (Insect. Soc)
Nannotrigona testaceicornis	2500	Brazil (ref: C. Menezes)	2016 Gruter etal (Insectes.Soc.) - suppl. C. Menezes and von Zuben, L. and Gruter, C. unpublished estimates
Nannotrigona testaceicornis	2500	Brazil (ref: C. Menezes)	2016 Gruter etal (Insectes.Soc.) - suppl. C. Menezes and von Zuben, L. and Gruter, C. unpublished estimates
Nannotrigona testaceicornis	2500	Brazil (ref: C. Menezes)	2016 Gruter etal (Insectes.Soc.) - suppl. C. Menezes and von Zuben, L. and Gruter, C. unpublished estimates
Nannotrigona testaceicornis	2500	Brazil (ref: C. Menezes)	2016 Gruter etal (Insectes.Soc.) - suppl. C. Menezes and von Zuben, L. and Gruter, C. unpublished estimates
Nannotrigona testaceicornis	2500	Brazil (ref: C. Menezes)	2016 Gruter etal (Insectes.Soc.) - suppl. C. Menezes and von Zuben, L. and Gruter, C. unpublished estimates
Nannotrigona testaceicornis	2500	Brazil (ref: C. Menezes)	2016 Gruter etal (Insectes.Soc.) - suppl. C. Menezes and von Zuben, L. and Gruter, C. unpublished estimates
Paratrigona subnuda	3500	Average colony size	2004 Tóth et al. (Insect. Soc)
Paratrigona subnuda	3500	Average colony size	2004 Tóth et al. (Insect. Soc)
Paratrigona subnuda	3500	Average colony size	2004 Tóth et al. (Insect. Soc)
Paratrigona subnuda	3500	Average colony size	2004 Tóth et al. (Insect. Soc)
Partamona helleri	5000	NA	NA
Partamona helleri	5000	NA	NA
Partamona helleri	5000	NA	NA
Partamona rustica	5000	NA	NA
Partamona rustica	5000	NA	NA
Partamona rustica	5000	NA	NA
Partamona rustica	5000	NA	NA
Partamona rustica	5000	NA	NA
Partamona rustica	5000	NA	NA
Partamona rustica	5000	NA	NA
Partamona rustica	5000	NA	NA
Partamona rustica	5000	NA	NA
Partamona rustica	5000	NA	NA
Partamona rustica	5000	NA	NA

Plebeia droryana	2960	Average colony size	2004 Tóth et al. (Insect. Soc)
Plebeia droryana	2960	Average colony size	2004 Tóth et al. (Insect. Soc)
Plebeia droryana	2960	Average colony size	2004 Tóth et al. (Insect. Soc)
Plebeia droryana	2960	Average colony size	2004 Tóth et al. (Insect. Soc)
Plebeia droryana	2960	Average colony size	2004 Tóth et al. (Insect. Soc)
Plebeia droryana	2960	Average colony size	2004 Tóth et al. (Insect. Soc)
Plebeia remota	2900	Average colony size	2004 Tóth et al. (Insect. Soc)
Scaptotrigona depilis	10375	NA	2014 Jaffé etal (Naturw.) ESM; 2017 Aleixo etal (Apidologie)
Scaptotrigona mexicana	2000	NA	2014 Jaffé etal (Naturw.) ESM
Scaptotrigona postica	15000	Average colony size	2004 Tóth et al. (Insect. Soc)
Schwarziana quadripunctata	1500	Average colony size	2004 Tóth et al. (Insect. Soc)
Tetragona clavipes	7000	Average colony size	2004 Tóth et al. (Insect. Soc)
Tetragonisca angustula	5000	Average colony size	2004 Tóth et al. (Insect. Soc)
Tetragonisca angustula	5000	Average colony size	2004 Tóth et al. (Insect. Soc)
Tetragonisca angustula	5000	Average colony size	2004 Tóth et al. (Insect. Soc)
Tetragonisca angustula	5000	Average colony size	2004 Tóth et al. (Insect. Soc)
Tetragonisca angustula	5000	Average colony size	2004 Tóth et al. (Insect. Soc)
Tetragonisca angustula	5000	Average colony size	2004 Tóth et al. (Insect. Soc)
Tetragonisca angustula	5000	Average colony size	2004 Tóth et al. (Insect. Soc)
Tetragonisca angustula	5000	Average colony size	2004 Tóth et al. (Insect. Soc)
Tetragonisca angustula	5000	Average colony size	2004 Tóth et al. (Insect. Soc)
Tetragonisca fiebrigi	5000	NA	NA
Trigona chanchamayoensis	500	NA	Contato pessoal Anderson Fernandes Miranda
Trigona fulviventris	8500	NA	2009 Oliveira (Acta.Amaz.); 1998 Slaa et al. (J. of Api. Res.); 2014 Jaffé etal (Naturw.) ESM
Trigona fulviventris	8500	NA	2009 Oliveira (Acta.Amaz.); 1998 Slaa et al. (J. of Api. Res.); 2014 Jaffé etal (Naturw.) ESM
Trigona hyalinata	40000	NA	Nieh et al 2013 (the royal society)
Trigona hyalinata	40000	NA	Nieh et al 2013 (the royal society)
Trigona spinipes	92500	Brazil (Itirapina - SP)	2014 Jaffé etal (Naturw.) ESM
Trigona spinipes	92500	Brazil (Itirapina - SP)	2014 Jaffé etal (Naturw.) ESM
Trigona spinipes	92500	Brazil (Itirapina - SP)	2014 Jaffé etal (Naturw.) ESM
Trigona spinipes	92500	Brazil (Itirapina - SP)	2014 Jaffé etal (Naturw.) ESM
Trigona spinipes	92500	Brazil (Itirapina - SP)	2014 Jaffé etal (Naturw.) ESM
Trigona spinipes	92500	Brazil (Itirapina - SP)	2014 Jaffé etal (Naturw.) ESM
Trigona spinipes	92500	Brazil (Itirapina - SP)	2014 Jaffé etal (Naturw.) ESM
Trigona spinipes	92500	Brazil (Itirapina - SP)	2014 Jaffé etal (Naturw.) ESM
Trigona williana	2492	French Guiana	1979 Roubik (J.Kansas.Entomol.Soc.)

Supplemental references

C. M. L. Aguiar, G. M. M. Santos, C. F. Martins, S. J. Presley, Trophic niche breadth and niche overlap in a guild of flower-visiting bees in a Brazilian dry forest. Apidologie. 44, 153–162 (2013).

C. M. L. Aguiar, Utilização de recursos florais por abelhas (Hymenoptera, Apoidea) em uma área de Caatinga (Itatim, Bahia, Brasil). Rev. Bras. Zool. 20, 457–467 (2003).

K. P. Aleixo, C. Menezes, V. L. Imperatriz-Fonseca, C. I. da Silva, Seasonal availability of floral resources and ambient temperature shape stingless bee foraging behavior (*Scaptotrigona aff. depilis*). Apidologie. 48, 117–127 (2017).

M. Alburaki, A. Gregorc, J. Adamczyk, and S. D. Stewart, Insights on Pollen Diversity of Honey Bee (*Apis Mellifera* L.) Colonies Located in Various Agricultural Landscapes. Southwest. Nat., 63, 1, 49–58 (2018), doi: 10.1894/0038-4909.63.49.

Y. Antonini, R. P. Martins, L. M. Aguiar, R. D. Loyola, Richness, composition and trophic niche of stingless bee assemblages in urban forest remnants. Urban Ecosyst. 16, 527–541 (2013).

F. M. Barbosa, R. M. de O. Alves, B. de A. Souza, & C. A. L. de Carvalho, Nest architecture of the stingless bee *Geotrigona subterranea* (Friese,1901) (Hymenoptera: Apidae: Meliponini). Biota Neotropica, 13(1), 147–152 (2013), https://doi.org/10.1590/s1676-06032013000100017

J. C. Biesmeijer, M. J. A. P. Smeets, J. A. P. Richter, M. J. Sommeijer, Nectar foraging by stingless bees in Costa Rica: Botanical and climatological influences on sugar concentration of nectar collected by Melipona. Apidologie. 30, 43–55 (1999).

S. Barroso-Arévalo, M. Vicente-Rubiano, J. A. Ruiz, A. Bentabol, and J. M. Sánchez-Vizcaíno, Does pollen diversity influence honey bee colony health?, Spanish J. Agric. Res., 17, 3 (2019), doi: 10.5424/sjar/2019173-13991.

A. M. Cavalcante, thesis, Universidade Federal do Ceará (2016).

A. P. A. da Cruz, M. C. Dórea, L. C. Lima e Lima, Pollen types used by *Centris (Hemisiella) tarsata* Smith (1874) (Hymenoptera, Apidae) in the provisioning of brood cells in an area of Caatinga. Acta Bot. Brasilica. 29, 282–284 (2015).

C. A. L. Carvalho, L. C. Marchini, P. B. Ros, Fontes de pólen utilizadas por *Apis mellifera* L. e algumas espécies de Trigonini (Apidae) em Piracicaba (SP). Bragantia. 58, 49–56 (1999).

E. Camillo, C. A. Garófalo, Analysis of the niche of two sympatric species of *Bombus* (Hymenoptera, Apidae) in southeastern Brazil. J. Trop. Ecol. 5, 81–92 (1989).

C M. Cortopassi-Laurino, A. Zillikens, J. Steiner, Pollen sources of the orchid bee *Euglossa annectans* Dressler 1982 (Hymenoptera: Apidae, Euglossini) analyzed from larval provisions. Genet. Mol. Res. 8, 546–556 (2009).

R. Cueva del Castillo, S. Sanabria-Urbán, M. A. Serrano-Meneses, Trade-offs in the evolution of bumblebee colony and body size: A comparative analysis. Ecol. Evol. 5, 3914–3926 (2015).

E. L. Franco, C. M. L. Aguiar, V. S. Ferreira, P. L. Oliveira-Rebouças, Plant use and niche overlap between the introduced honey bee (Apis mellifera) and the native bumblebee (*Bombus atratus)* (Hymenoptera: Apidae) in an area of tropical mountain vegetation in northeastern Brazil. Sociobiology. 53, 141–150 (2009).

L. B. Faria, thesis, Universidade de São Paulo (2014).

M. G. Ferreira, M. L. Absy, and A. C. C. Rezende, Pollen collected and trophic interactions between stingless bees of the genera *Melipona*, *Frieseomelitta* and *Plebeia* (Apidae: Meliponini) raised in Central Amazon. J. Apic. Res., 1–13 (2021), doi: 10.1080/00218839.2021.1898837.

M. G. Ferreira, M. L. Absy, Pollen niche and trophic interactions between colonies of *Melipona (Michmelia) seminigra merrillae* and *Melipona (Melikerria) interrupta* (Apidae: Meliponini) reared in floodplains in the Central Amazon. Arthropod. Plant. Interact. 9, 263–279 (2015).

A. Guzman et al., Surrounding landscape and spatial arrangement of honey bee hives affect pollen foraging and yield in cranberry. Agric. Ecosyst. Environ., 286, no. August (2019), doi: 10.1016/j.agee.2019.106624.

C. Grüter, L. G. von Zuben, F. H. I. D. Segers, J. P. Cunningham, Warfare in stingless bees. Insectes Soc. (2016), doi:10.1007/s00040-016-0468-0.

D. Goulson, W. O. H. Hughes, L. C. Derwent, J. C. Stout, Colony growth of the bumblebee, *Bombus terrestris*, in improved and conventional agricultural and suburban habitats. Oecologia. 130, 267–273 (2002).

G L. F. Gostinski, thesis, Universidade Federal do Maranhão (2018).

S. D. Hilário, V. L. Imperatriz-Fonseca, Pollen foraging in colonies of *Melipona bicolor* (Apidae, Meliponini): effects of season, colony size and queen number. Genet. Mol. Res. 8, 664–671 (2009).

R. Jaffé, F. C. Pioker-Hara, C. F. Santos, L. R. Santiago, D. A. Alves, A. M. P. Kleinert, T. M. Francoy, M. C. Arias, V. L. Imperatriz-Fonseca, Monogamy in large bee societies: a stingless paradox. Naturwissenschaften. 101, 261–264 (2014).

A. Koetz, Ecology, behaviour and control of *Apis cerana* with a focus on relevance to the Australian incursion. Insects. 4, 558–592 (2013).

R. Kajobe, Pollen foraging by Apis mellifera and stingless bees *Meliponula bocandei* and *Meliponula nebulata* in Bwindi Impenetrable National Park, Uganda. Afr. J. Ecol. 45, 265–274 (2006).

W. E. Kerr, M. Petrere Jr., J. A. F. Diniz-Filho, Informações biológicas e estimativa do tamanho ideal da colmeia para a abelha tiúba do Maranhão (*Melipona compressipes fasciculata* Smith - Hymenoptera, Apidae). Rev. Bras. Zool. 18, 45–52 (2001).

C. I. S. Lucas, W. C. Andrade, A. F. Ferreira, G. S. Sodré, C. A. L. Carvalho, M. A. P. C. Costa, C. M. L. Aguiar, Pollen types from colonies of *Melipona scutellaris* Latreille, 1811 (Hymenoptera: Apidae) established in a coffee plantation. Grana. 57, 235–245 (2017).

I. N. Lima, thesis, Universidade Federal do Ceará (2017).

M. Lindauer, W. E. Kerr, Communication between the workers of stingless bees. Bee World. 41, 29–41; 65–71 (1960).

S. P. Lawson, K. N. Ciaccio, S. M. Rehan, Maternal manipulation of pollen provisions affects worker production in a small carpenter bee. Behav. Ecol. Sociobiol. 70, 1891–1900 (2016).

Y. J. Liu, T. R. Zhao, X. W. Zhang, C. Liang, F. Y. Zhao, Melittopalynology and trophic niche analysis of *Apis cerana* and *Apis mellifera* in Yunnan province of southwest China. Sociobiology. 60, 289–294 (2013).

C. Maia-Silva, V. L. Imperatriz-Fonseca, C. I. Silva, M. Hrncir, Environmental windows for foraging activity in stingless bees, *Melipona subnitida* Ducke and *Melipona quadrifasciata* Lepeletier (Hymenoptera: Apidae: Meliponini). Sociobiology. 61, 378–385 (2014).

E. A. Miranda, thesis, Universidade Federal de São Carlos (2016).

T. Machado, B. F. Viana, C. I. da Silva, and D. Boscolo, How landscape composition affects pollen collection by stingless bees?. Landsc. Ecol., 35, 3, 747–759 (2020), doi: 10.1007/s10980-020-00977-y.

J. C., Nieh, F. A. L., Contrera and P., Nogueira–Neto, Pulsed mass recruitment by a stingless bee, *Trigona hyalinata*. Proc. R. Soc. Lond. B.2702191–2196 (2003).

F. P. M. Oliveira, M. L. Absy, I. S. Miranda, Recurso polínico coletado por abelhas sem ferrão (Apidae, Meliponinae) em um fragmento de floresta na região de Manaus - Amazonas. Acta Amaz. 39, 505–518 (2009).

F. P. M. Oliveira, thesis, Instituto Nacional de Pesquisas da Amazônia (2003).

R. S. Pinto, A. G. Silva, M. M. C. Rêgo, and P. M. C. Albuquerque, Pollen analysis of the post-emergence residue of *Euglossa* bees (Apidae: Euglossini) nesting in an urban fragment. Sociobiology, 66, 1, 88–96 (2019) doi: 10.13102/sociobiology.v66i1.3434.

A. D. Ritchie, R. Ruppel, S. Jha, Generalist behavior describes pollen foraging for perceived oligolectic and polylectic bees. Environ. Entomol. 45, 909–919 (2016).

D. W. Roubik, Nest and colony characteristics of stingless bees from French Guiana (Hymenoptera: Apidae). J. Kansas Entomol. Soc. 52, 443–470 (1979).

L. S. Rabelo, A. M. G. F. Vilhena, E. M. A. F. Bastos, C. M. L. Aguiar, S. C. Augusto, Oil-collecting bee–flower interaction network: do bee size and anther type influence the use of pollen sources? Apidologie. 46, 465–477 (2015).

A. Saravia-Nava, H. M. Niemeyer, and C. F. Pinto, Pollen Types Used by the Native Stingless Bee, *Tetragonisca angustula* (Latreille), in an Amazon-Chiquitano Transitional Forest of Bolivia. Neotrop. Entomol., 47, 6, 798–807 (2018), doi: 10.1007/s13744-018-0612-9.

C. F. Santos, M. L. Absy, Polinizadores de *Bertholletia excelsa* (Lecythidales: Lecythidaceae): Interações com abelhas sem ferrão (Apidae: Meliponini) e nicho trófico. Neotrop. Entomol. 39, 854–861 (2010).

C. I. Silva, C. M. Hirotsu, A. J. de S. P. Filho, E. P. Queiroz, C. A. Garófalo, Is the maximum reproductive rate of *Centris analis* (Hymenoptera, Apidae, Centridini) associated with floral resource availability? Arthropod. Plant. Interact. 11, 389–402 (2017).

E. J. Slaa, A. Cevaal, M. J. Sommeijer, Floral constancy in *Trigona* stingless bees foraging on artificial flower patches: A comparative study. J. Apic. Res. 37, 191–198 (1998).

G. Suwannapong, M. E. Benbow, and J. C. Nieh, Biology of thai honeybees: natural history and threats. Landscape Ecol., 1–327 (2012).

W. O. Sabino, E. M. A. F. Bastos, Y. Antonini, Trophic-niche of the Leaf Cutter Bee *Megachile (Moureapis) maculata* (Hymenoptera: Megachilidae) in Southeastern Brazil. J. Kansas Entomol. Soc. 89, 373–381 (2016).

W. O. Sabino, I. Alves, C. I. da Silva, Versatility of the trophic niche of *Centris (Paracentris) burgdorfi*. Arthropod. Plant. Interact. 13, 227–237 (2019).

E. Tóth, D. C. Queller, A. Dollin, J. E. Strassmann, Conflict over male parentage in stingless bees. Insectes Soc. 51, 1–11 (2004).

F. G. Vossler, thesis, Universidad Nacional de la Plata (2012).

P. Vit, S. R. M. Pedro, D. W. Roubik, Pot-pollen in stingless bee melittology. Springer International Publishing. (2018).

W. Wilms, I.-F. V. L., W. Engels, Resource partitioning between highly eusocial bees and possible impact of the introduced africanized honey bee on native stingless bees in the brazilian atlantic rainforest. Stud Neotrop Fauna Environm. 31, 137–151 (1996).
